# Discovery of Secondary Metabolites from the Sponge-Derived Fungus *Aspergillus templicola*

**DOI:** 10.3390/md23070285

**Published:** 2025-07-09

**Authors:** Kai Li, Yue Zhang, Lei Li, Sen Wang, Cili Wang, Pinglin Li

**Affiliations:** 1 Key Laboratory of Marine Drugs, Chinese Ministry of Education, School of Medicine and Pharmacy, Ocean University of China, Qingdao 266003, China; 17854232177@163.com (K.L.); zhangyue_00803@163.com (Y.Z.); ll1061602375@163.com (L.L.); wangsen19972021@163.com (S.W.); 2 Laboratory for Marine Drugs and Bioproducts, Qingdao Marine Science and Technology Center, Qingdao 266237, China; 3 Key Laboratory of Marine Food Processing & Safety Control, College of Food Science and Engineering, Ocean University of China, Qingdao 266003, China

**Keywords:** *Aspergillus templicola*, cytochalasins, cyclic pentapeptides, anti-inflammatory activity, cytotoxic activity

## Abstract

Combining biosynthetic gene cluster analysis with the OSMAC strategy, fractionation of the fermentation extract of *Aspergillus templicola* from the sponge *Agelas* sp. led to the isolation of four novel cytochalasins, colachalasins J–M (**1**–**4**), a novel cyclic pentapeptide, avellanin P (**5**), together with five known compounds (**6**–**10**). The structures of **1**–**9** were elucidated using spectroscopic data, single crystal X-ray diffraction, and Marfey’s analysis. Compound **2** exhibited potent anti-inflammatory activity in zebrafish assays. Additionally, Compounds **4** and **6** showed modest cytotoxicity against several human cancer cell lines with IC_50_ values ranging from 2.6 to 11.2 μm.

## 1. Introduction

Marine-derived fungi are prominent producers of novel bioactive compounds and drug lead compounds [1]. With the advances in sequencing technologies, genomic data have emerged as a valuable resource containing unique biosynthetic gene clusters (BGCs), enabling the prediction of compound types, and facilitating the discovery of novel natural products (NPs) [2]. However, under traditional laboratory conditions, fungi transcribe only a small fraction of their biological genetic clusters, while most genes remain silent [3]. Consequently, traditional cultivation methods can access only a limited portion of the potential chemical diversity of fungal natural products [4]. Numerous strategies aim to activate these biosynthetic gene clusters to broaden metabolite diversity. One such approach is the One Strain Many Compounds (OSMAC) method, which diversifies culture media and growth conditions to generate environmental cues, induce silent biosynthetic clusters, and accumulate cryptic metabolites [5]. Co-cultivation and epigenetic modification represent other important components of the OSMAC approach [6,7].

**Figure 1:**
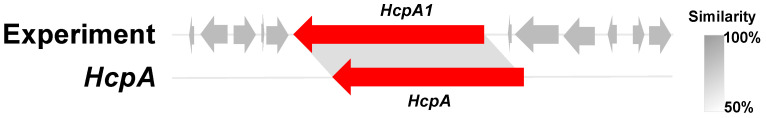
The *HcpA1* gene cluster in comparison to *HcpA*.

**Figure 2:**
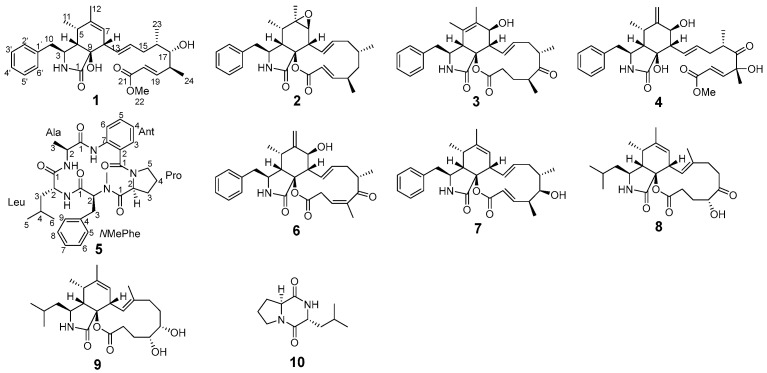
Structures of compounds **1**–**10**.

During the early stages of our research, a partial gene cluster (*cck*) was discovered in *Aspergillus templicola*, isolated from the sponge *Agelas* sp. [8]. This *cck* gene cluster exhibits similarity to the previously reported *ccs* gene cluster [9], suggesting its potential for synthesizing cytochalasins. Our recent chemical investigations of the sponge-derived fungus *A. templicola* cultured in liquid medium successfully yielded 13 cytochalasins, including 9 novel cytochalasins, colachalasins (A–I), and 4 known cytochalasins [8]. Furthermore, genomic analysis revealed another *par* gene cluster homologous to the previously reported *hcpA* cluster (Figure 1) [10], indicating its potential role in cyclic peptide biosynthesis. Cyclic peptides have attracted significant attention due to their unique biological activities, such as cytotoxicity [11], antimalarial activity [12], and antibacterial activity [13]. However, cyclic peptides were not found in our previous research. To activate cryptic biosynthetic pathways for cyclic peptides, an OSMAC strategy was employed by switching to a rice-based solid medium. Ultimately, this process led to the isolation of four novel cytochalasins, colachalasins J–M (**1**–**4**), a novel cyclic pentapeptide, avellanin P (**5**), along with five known compounds, cytochalasin Z_16_ (**6**) [14], 7-Deoxy-cytochalasin Z_7_ (**7**) [15], aspochalasin I (**8**) [16], aspochalasin A_1_ (**9**) [17], and cyclo-(*S*-Pro-*R*-Leu) (**10**) [18] (Figure 2). Herein, we report the isolation, structural elucidation, and biological activities of the new isolates.

## 2. Results

Colachalasin J (**1**) was obtained as colorless crystals. The molecular formula was determined to be C_29_H_39_NO_5_ based on the [M + H]^+^ ion peak at m/z 482.2892 [M + H]^+^ (calcd for 482.2901) in HRESIMS, requiring eleven degrees of unsaturation. The ^1^H NMR data (Table S2) indicated the presence of four methyl groups, a methoxy group, three double bonds, and a monosubstituted phenyl group. The ^13^C NMR data (Table S2) and the HSQC spectrum revealed 29 carbon signals, which were categorized into five methyl groups, two methylene groups, seventeen methine carbons (ten olefinic), and five non-hydrogenated carbons (two of which were carbonyls). These NMR data indicated that compound **1** is a cytochalasin [8].

**Figure 3:**
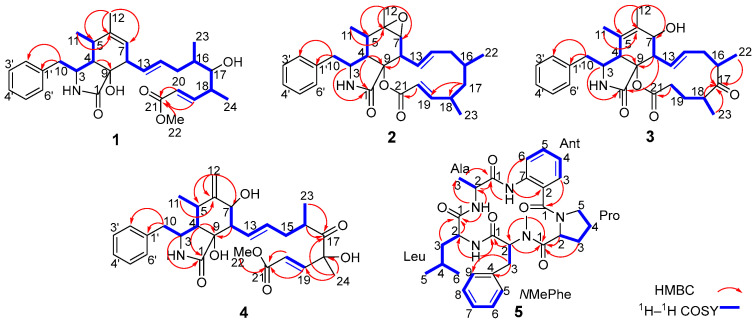
The Key ^1^H–^1^H COSY, HMBC correlations of compounds **1**–**5**.

Analysis of the 1D and 2D NMR data (Table S2 and Figure 3) revealed that the structure of compound **1** is similar to that of compound **7** (Figure 2), a cytochalasin previously isolated from a marine-derived fungus *Spicaria elegans* [15], with the main difference being the ester bond between C-9 and C-21 of **7** is cleaved in **1**, to give the open methyl ester. Compared with **7**, the variation is that of an additional hydroxy group at C-9, and a methoxy group at C-22 in **1**. These structural differences are further confirmed by the HMBC correlations from H-19, H-20, and H_3_-22 to C-21 (Figure 3), and from H-4 to C-9 (*δ*_C_ 80.2), which is consistent with the molecular formula.

**Figure 4:**
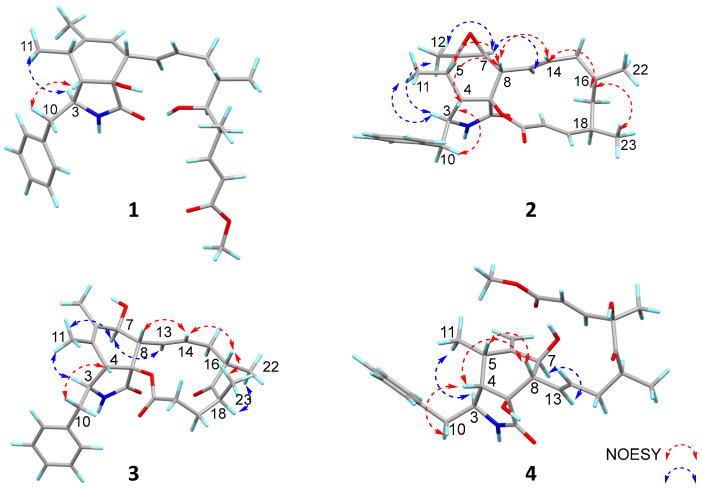
The Key NOESY correlations of compounds **1**–**4**.

**Figure 5:**
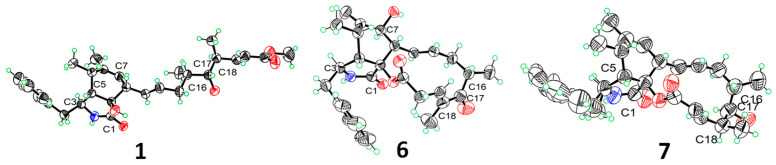
X-ray crystal structures of **1**, **6**, **7**. The ellipsoids of non-hydrogen atoms of **1**, **6**, **7** are shown at 50% probability levels.

In the NOESY spectrum of compound **1** (Figure 4), the correlation between H-3 and H_3_-11 indicated their co-facial orientation, which was assigned as *α*-orientation. The correlations of H_2_-10/H-4 confirmed the *β*-orientation of these protons. The large coupling constants (*J*_13,14_ = 15.2 Hz; *J*_19,20_ = 15.8 Hz) indicated that the Δ^13^ and Δ^19^ double bonds were both *trans* configured. The determination of the relative configuration at C-8, C-9, C-16, C-17, and C-18 was challenging on account of the absence of effective NOESY correlations. Fortunately, suitable single crystals were acquired and subjected to a Bruker APEX-II CCD diffractometer with Cu K*α* radiation, determining the absolute configuration of compound **1** as 3*S*, 4*S*, 5*S*, 8*S*, 9*R*, 16*S*, 17*S*,18*S*, with a Flack parameter of 0.03 (11) (Figure 5).

Colachalasin K (**2**) was obtained as a colorless powder. The molecular formula was determined to be C_28_H_35_NO_4_ based on the [M + H]^+^ ion peak at *m*/*z* 450.2631 (calcd for 450.2639) in HRESIMS. The 1D NMR data (Table S2) indicated that the structure of compound **2** were similar to those of colachalasin D (Figure S1), a cytochalasin previously isolated from the fungus *A. templicola* [8]. The key difference between them were the absence of a 17-carbonyl group and the an additional Δ^19^ double bond in compound **2**, as confirmed by the ^1^H–^1^H COSY correlations of H-16/H-17/H-18 (Figure 3), the HMBC correlations of H-17/C-18, H-17/C-19, H-20/C-21 (Figure 3), and the chemical shifts of C-17 (*δ*_C_ 44.8), C-19 (*δ*_C_ 161.9), and C-20 (*δ*_C_ 118.9). Thus, the planar structure of compound **2** was established.

**Figure 6:**
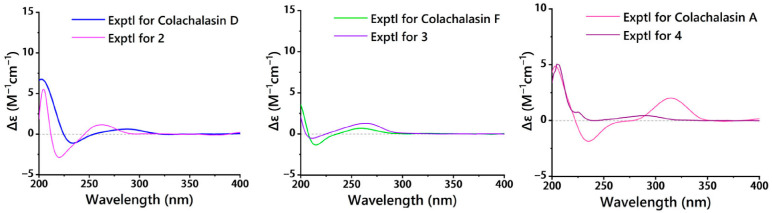
ECD spectra of **2** and colachalasin D, **3** and colachalasin F, **4** and colachalasin A.

The relative configuration of compound **2** was determined by the analysis of the NOESY spectrum (Figure 4). The correlations of H-3/H_3_-11, H-3/H_3_-12, H_3_-12/H-7, H-7/H-13 suggested the *α*-orientation of H-3, H_3_-11, H_3_-12, H-7. Additionally, the NOESY correlation of H-5/H-8, and H-4/H_2_-10, H-4/H-8, H-8/H-14, H-14/H-16, H-16/H_3_-23, suggested the *β*-orientation of H-4, H-5, H-8, H-16, H_3_-23. Previous studies have suggested that the core structural elements of cytochalasins mostly share a conserved stereochemical configuration, and that is the 5/6 ring junction and the trans-stereochemistry of the macrocyclic ring [19]. This stereochemical assignment is further corroborated by X-ray crystallographic data of cytochalasins [8]. Consequently, the oxygen atom at C-9 is *β*-oriented. The large coupling constants (*J*_13, 14_ = 15.5 Hz; *J*_19, 20_ = 15.9 Hz) indicated that the Δ^13^ and Δ^19^ double bonds were both *trans* configured. The absolute configurations of compound **2** were assigned as 3*S*, 4*S*, 5*S*, 6*R*, 7*S*, 8*S*, 9*S*, 16*S*, 18*R* by comparison of their similar ECD spectra between **2** and colachalasin D (Figure 6).

Colachalasin L (**3**), obtained as colorless powder, was determined to have the molecular formula C_28_H_35_NO_5_ based on the HRESIMS ion at *m*/*z* 464.2430 (calcd for 464.2442) [M − H]^−^. The ^1^H and ^13^C NMR data (Table S3) indicated that the structure of compound **3** was similar to colachalasin F (Figure S1), a cytochalasin previously isolated from the fungus *A*. *templicola* [8], with the exception of an additional 17-carbonyl group and the absence of the Δ^19^ double bond in compound **3** compared to colachalasin F. This was further confirmed by the HMBC correlations of H-16, H-18, H_3_-22, H_3_-23/C-17 (*δ*_C_ 216.8) (Figure 3) and the chemical shifts of C-19 (*δ*_C_ 29.3) and C-20 (*δ*_C_ 33.2).

The relative configuration of the stereocenters and the configuration of the double bond in compound **3** were determined by analysis of NOESY data and coupling constants (Figure 4 and Table S3). In the NOESY spectrum, correlations of H-3/H_3_-11, H_3_-11/H-7, H-7/H-13 suggested the *α*-orientation of H-3, H-7. The correlations of H-4/H_2_-10, H-8/H-14, H-14/H-16, H-16/H_3_-23 suggested the *β*-orientation of H-4, H-8, H-16, H_3_-23. The large coupling constants (*J*_13,14_ = 15.4 Hz) indicated that the Δ^13^ double bond was *trans* configured. Based on a comparison of ECD spectra (Figure 6) between **3** and colachalasin F, which showed similar structures, the absolute configuration of compound **3** was assigned as 3*S*, 4*S*, 7*S*, 8*S*, 9*S*, 16*S*, 18*S*.

Colachalasin M (**4**) was isolated as a colorless oil. The molecular formula was determined to be C_29_H_37_NO_7_ based on the HRESIMS data (*m*/*z* 512.2653 [M + H]^+^, calcd for 512.2643). The 1D NMR data (Table S3) of compound **4** were similar to those of colachalasin A (Figure S1), a cytochalasin previously isolated from the fungus *A*. *templicola* [8]. The only notable difference was that of the presence of a disubstituted terminal double bond at C-6 in **4**, which was replaced by the 6, 7-epoxide ring in colachalasin A. These differences are further supported by the HMBC correlations of H_2_-12/C-5 (*δ*_C_ 31.6), H_2_-12/C-6 (*δ*_C_ 148.9), and H_2_-12/C-7 (*δ*_C_ 70.2) (Figure 3).

The relative configuration of compound **4** was determined based on NOESY correlations (Figure 4). In the NOESY spectrum, correlations between H-3/H_3_-11, H-7/H-13, and H-4/H_2_-10, H-4/H-8, H-5/H-8 suggested the α-orientation of H-3, H_3_-11, H-7, and the β-orientation of H-4, H-5, H-8. The large coupling constants (*J*_13,14_ = 15.3 Hz; *J*_19,20_ = 15.8 Hz) indicated that the Δ^13^ and Δ^19^ double bonds were both *trans* configured. The stereochemical configuration of the long carbon chain in compound **4** was deduced from the NMR data. The nearly identical NMR data for the chiral centers of the side chain in compounds **4** and colachalasin A suggest that the relative configuration of the side chain in compound **4** is the same as in colachalasin A. A comparison of ECD spectra between **4** and colachalasin A (Figure 6) determined the absolute configuration of compound **4** as 3*S*, 4*S*, 5*S*, 7*S*, 8*S*, 9*S*, 16*S*, 18*R.*

Compound **5** was obtained as a yellow oil. The molecular formula, C_31_H_39_N_5_O_5_, was determined based on HRESIMS data (*m*/*z* 562.3037 [M + H]^+^, calcd for 562.3024), implying fifteen degrees of unsaturation. The ^1^H NMR data (Table S4) and HSQC spectrum indicated the presence of three amide protons (*δ*_H_ 9.30, *δ*_H_ 7.01, and *δ*_H_ 7.54) and one *N*-methyl group (*δ*_H_ 2.01). The ^13^C NMR data (Table S4) and HSQC spectrum revealed the presence of 31 carbons, including 8 non-protonated carbons (2 olefinic and 5 carbonyl), 14 methines (5 sp^3^ hybridized and 9 olefinic), 5 methylenes (all sp^3^ hybridized), and 4 methyls (all sp^3^ hybridized).

Further analysis of 2D NMR spectra (Figures S38–S40) led to the identification of five amino-acid components. Alanine (Ala) was confirmed by the ^1^H−^1^H COSY correlations for H-2 (*δ*_H_ 5.19)/H_3_-3 (*δ*_H_ 1.52), together with the HMBC correlations for H-2, H_3_-3/C-1 (*δ*_C_ 171.2), NH (*δ*_H_ 7.54)/C-2, and NH (*δ*_H_ 7.54)/C-3 (Figure 3). The anthranilic acid unit (Ant) was confirmed by the ^1^H−^1^H COSY correlations for a spin system consisting of four adjacent aromatic protons (*δ*_H_ 6.85, 6.76, 7.03, and 8.57), together with the HMBC correlations for H-3/C-1(*δ*_C_ 169.0) and NH (*δ*_H_ 9.30)/C-2, NH (*δ*_H_ 9.30)/C-6. Another aliphatic amino acid, proline (Pro), was deduced from sequential ^1^H−^1^H COSY correlations for H-2 (*δ*_H_ 3.97)/H_2_-3 (*δ*_H_ 1.17)/H_2_-4 (*δ*_H_ 1.43/1.03)/H_2_-5 (*δ*_H_ 2.70) and the HMBC correlations for H-2, H_3_-3/C-1(*δ*_C_ 172.2). An *N*Me-phenylalanine unit (*N*Me-Phe) was confirmed by the HMBC correlations of an *N*Me (*δ*_H_ 2.01)/C-2 (*δ*_C_ 69.4), the ^1^H−^1^H COSY correlations of H-2 (*δ*_H_ 3.47)/H_2_-3 (*δ*_H_ 3.67/3.87), and a phenyl ring assigned at C-3 by sequential ^1^H−^1^H COSY correlations of five CH (*δ*_H_ 6.91, 7.02) protons and the HMBC correlations of H-3/C-4, H-3/C-9. The last unit was assigned as leucine (Leu) by the ^1^H−^1^H COSY correlations for H-2 (*δ*_H_ 5.10)/H_2_-3 (*δ*_H_ 2.41/2.01)/H-4 (*δ*_H_ 1.89)/H_3_-5 (*δ*_H_ 0.93), and H-4/H-6 (*δ*_H_ 0.97), together with the HMBC correlations for H-2, H_2_-3/C-1 (*δ*_C_ 171.7), and NH (*δ*_H_ 7.01)/C-2.

The connectivity among the five amino acid residues was determined by the HMBC correlations from Ant-NH to Ala-C1, AlaNH to Leu-C1, Leu-NH to *N*Me-Phe-C1, and *N*Me-Phe-CH_3_ to Pro-C1 (Figure 3). The correlations between Ant to Pro was established based on HRESIMS data (*m/z* 562.3037 [M + H]^+^, calcd for 562.3024), implying fifteen degrees of unsaturation. Finally, the Marfey’s analysis confirmed the absolute configurations of each amino acid (Figure S51). Thus, compound **5** was determined as *cyclo* (Ant^1^-_L_-Pro^2^-*N*Me-_L_-Phe^3^-_D_-Leu^4^-_L_-Ala^5^).

Furthermore, the known compounds **6** and **7** were crystallized from a mixed solvent of ethanol and H_2_O, and their single-crystal X-ray diffraction analysis is shown in Figure 5.

**Figure 7:**
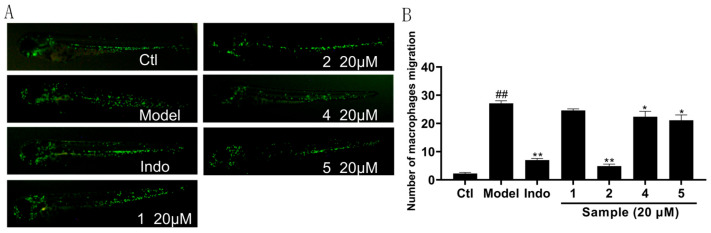
Anti-inflammatory effect of compounds **1, 2, 4, 5.** (**A**) Inflammatory areas in transgenic fluorescent zebrafish (Tg: *zlyz-EGFP*) generated by CuSO_4_ that express enhanced green fluorescent protein after being treated with compounds **1, 2**, **4**, **5.** (**B**) Quantitative evaluation of fluorescent macrophage counts in the vicinity of inflammatory sites in zebrafish treated with compounds **1, 2**, **4**, **5**. The results were subjected to analysis using one-way analysis of variance followed by Dunnett’s post hoc *t*-test. *^##^ p* ≤ 0.01 vs. Ctl, * *p* ≤ 0.05 vs. model, ** *p* ≤ 0.01 vs. Model.

Considering the remaining amount of these compounds, compounds **1**, **2**, **4**, **5** were selected for evaluation of anti-inflammatory activity in CuSO_4_-induced transgenic fluorescent zebrafish. The extent of inflammation was assessed by monitoring the migration of macrophages along the lateral line. As shown in Figure 7A, compounds **2**, **4**, and **5** reduced inflammatory, as evidenced by a higher accumulation of macrophages at the lateral line compared to controls. Furthermore, as shown in Figure 7B, compound **2** at 20 μm demonstrated superior anti-inflammatory activity compared to the positive control indomethacin at 20 μm.

The cytotoxicity of the isolated compounds **1**–**2** and **4**–**7** was evaluated against human leukemia K562, human pancreatic cancer ASPC-1, human breast cancer MDA-MB-231, and human small cell lung cancer NCI-H446/NCI-H446/EP cell lines. As shown in Table 1, compounds **4** and **6** exhibited moderate cytotoxicity against NCI-H446 cell lines with IC_50_ values of 4.2 and 2.6 μm, respectively, as well as against NCI-H446/EP cell lines with IC_50_ values of 6.5 and 11.2 μm, respectively. The IC_50_ value of compound **4** against MDA-MB-231 cells was 9.0 μm. Other compounds were weakly active or totally inactive against the human cancer cell lines.

## 3. Materials and Methods

### 3.1. General Experimental Procedures

Optical rotations were measured using a Jasco P-1020 digital polarimeter (Jasco, Tokyo, Japan). Ultraviolet (UV) spectra were recorded on a Beckman DU640 spectrophotometer (Brea, CA, USA). Electronic circular dichroism (ECD) spectra were acquired using a Jasco J-810 spectropolarimeter (Jasco, Tokyo, Japan). NMR spectra were obtained on an Agilent DD2-500 (^1^H, 500 MHz; ^13^C, 125 MHz; Agilent, Beijing, China). For NMR spectra in CDCl_3_, the residual CHCl_3_ resonance (δ_H_ 7.26 ppm) and the CDCl_3_ resonance (δ_C_ 77.16 ppm) served as internal references for ^1^H and ^13^C NMR, respectively. Similarly, in CD_3_OD, the residual CH_3_OH resonance (δ_H_ 3.31 ppm) and the CD_3_OD resonance (δ_C_ 49.00 ppm) were used as internal standards. For NMR spectra in C_6_D_6_, the residual C_6_D_6_ resonance (*δ*_H_ 7.16 ppm) and the C_6_D_6_ resonance (*δ*_C_ 128.1 ppm) were used as internal standards. High-resolution electrospray ionization mass spectrometry (HRESIMS) data were collected on a Micromass Q-Tof Ultima GLOBAL GAA076LC mass spectrometer (Autospec-Ultima-TOF, Waters, Shanghai, China). Semi-preparative HPLC was performed using a Waters 1525 pump equipped with a 2998 photodiode array detector and a YMC C18 column (10 × 250 mm, 5 μm). Column chromatography was carried out using silica gel (200–300 mesh, 300–400 mesh, or H grade). Melting points were determined on a ZGX-5 Plus microscopic melting point apparatus (Shanghai Zhuoguang Instrument Technology Co., Ltd., Shanghai, China).

### 3.2. Fungus Material

The fungal strain 18XS-01-ZM-05, isolated in July 2018 from the South China Sea sponge *Agelas* sp. (Xisha Island [Yagong Island]; 16°34′N, 111°41′E), was identified as *A*. *templicola* through ITS sequence analysis (GenBank PQ685989), which showed 99% identity to the corresponding sequence of *A*. *templicola* (GenBank OL711823.1). The strain was deposited at the Key Laboratory of Marine Drugs, Chinese Ministry of Education, Ocean University of China.

### 3.3. Fermentation, Extraction, and Isolation

Solid-state fermentation was conducted in 100 flasks, each containing a sterile medium of rice (100 g), glucose (2 g), NaCl (1 g), and distilled water (100 mL). *A. templicola* was aseptically inoculated into each flask. After 56 days of fermentation, the fermentations were all combined and the supernatant was extracted three times with EtOAc.

The ethyl acetate extract (96.2 g) was fractionated by a silica gel column chromatography (CC) eluted with sequential gradient elution petroleum ether/acetone (from 100:1 to 1:1, *v*/*v*), then eluted with a gradient of CH_2_Cl_2_/CH_3_OH (from 20:1 to 1:1, *v*/*v*) to obtain eight fractions.

Fr.4 (2.8 g) was subjected to silica gel CC (petroleum ether/acetone, from 30:1 to 1:1, *v/v*), yielding six subfractions (Fr.4.1–Fr.4.6). Fr.4.5 was further purified by silica gel CC (petroleum ether/acetone, from 20:1 to 1:1, *v/v*), resulting in six subfractions (Fr.4.5.1–Fr.4.5.6). Fr.4.5.3 was purified using semi-preparative HPLC (ODS, 5 μm, 250 × 10 mm; CH_3_OH/H_2_O, 75:25, *v*/*v*; 2 mL·min^−1^) to yield **1** (2.2 mg).

Fr.5 (3.1 g) was subjected to silica gel CC (petroleum ether/acetone, from 25:1 to 1:1, *v*/*v*) and divided into nine subfractions (Fr.5.1–Fr.5.9). Fr.5.2 was purified by silica gel CC (petroleum ether/acetone, from 25:1 to 1:1, *v*/*v*), resulting in six subfractions (Fr.5.2.1–Fr.5.2.6). Fr.5.2.4 was purified using semi-preparative HPLC (ODS, 5 μm, 250 × 10 mm; CH_3_OH/H_2_O, 70:30, *v*/*v*; 2 mL·min^−1^) to yield **8** (21.6 mg), **9** (22.2 mg). Fr.5.6 was separated by silica gel CC (petroleum/acetone, from 15:1 to 1:1, *v*/*v*) into five subfractions (Fr.5.6.1–Fr.5.6.5). Fr.5.6.2 was purified by semi-preparative HPLC (ODS, 5 μm, 250 × 10 mm; CH_3_OH/H_2_O, 68:32, *v*/*v*; 2 mL·min^−1^) to afford **5** (2.2 mg).

Fr.6 (2.5 g) was separated by silica gel CC (petroleum/acetone, from 20:1 to 1:1, *v*/*v*) into eight subfractions (Fr.6.1–Fr. 6.8). Fr.6.2 was subjected to silica gel CC (petroleum/acetone, from 20:1 to 1:1, *v*/*v*), yielding nine fractions (Fr.6.2.1–Fr.6.2.9). Fr.6.2.2 was purified using semi-preparative HPLC (ODS, 5 μm, 250 × 10 mm; CH_3_OH/H_2_O, 65:35, *v*/*v*; 2 mL·min^−1^) to yield **7** (14.5 mg). Fr.6.8 was separated by silica gel CC (petroleum/acetone, from 15:1 to 1:1, *v*/*v*) into five fractions (Fr.6.8.1–Fr.6.8.5). Fr.6.8.5 was purified by semi-preparative HPLC (ODS, 5 μm, 250 × 10 mm; CH_3_CN/H_2_O, 45:55, *v*/*v*; 2 mL·min^−1^) to afford **4** (3.8 mg).

Fr.7 (5.1 g) was separated by silica gel CC (petroleum/acetone, from 15:1 to 1:1, *v*/*v*) into nine subfractions (Fr.7.1–Fr. 7.9). Fr.7.2 was subjected to silica gel CC (petroleum/acetone, from 15:1 to 1:1, *v*/*v*), yielding five fractions (Fr.7.2.1–Fr.7.2.5). Fr.7.2.3 was purified using semi-preparative HPLC (ODS, 5 μm, 250 × 10 mm; CH_3_OH/H_2_O, 65:35, *v*/*v*; 2 mL·min^−1^) to yield **2** (2.6 mg). Fr.7.7 was separated by silica gel CC (petroleum/acetone, from 10:1 to 1:1, *v*/*v*) into six fractions (Fr.7.7.1–Fr.7.7.6). Fr.7.7.4 was purified by semi-preparative HPLC (ODS, 5 μm, 250 × 10 mm; CH_3_CN/H_2_O, 40:60, *v*/*v*; 2 mL·min^−1^) to afford **3** (4.4 mg) and **6** (6.5 mg).

Fr.8 (8.3 g) was subjected to silica gel CC (petroleum ether/acetone, from 10:1 to 1:1, *v*/*v*), yielding nine subfractions (Fr.8.1–Fr.8.9). Fr.8.5 was further purified by silica gel CC (petroleum ether/acetone, from 10:1 to 1:1, *v*/*v*), resulting in six subfractions (Fr.8.5.1–Fr.8.5.6). Fr.8.5.2 was purified using semi-preparative HPLC (ODS, 5 μm, 250 × 10 mm; CH_3_OH/H_2_O, 50:50, *v*/*v*; 2 mL·min^−1^) to yield **10** (86.3 mg).

Colachalasin J (**1**)

Colorless crystal; mp 160.1–164.2 °C; [*α*]_D_^25^ + 30.2 (*c* 0.2, CH_3_OH); UV (CH_3_OH) *λ*_max_ (log *ε*) 196 (1.67), 214 (0.75), 250 (0.09) nm; ECD (0.25 mm, CH_3_OH) *λ*_max_ (Δ*ε*) 206 (4.73), 229 (–1.16), 258 (0.07) nm; IR (KBr) *ν*_max_ 2969, 2361, 2335, 1699, 1540, 1454, 1268, 1100 cm^−1^; ^1^H and ^13^C NMR data see Table S2; HRESIMS *m*/*z* 482.2892 [M + H]^+^ (calcd for C_29_H_40_NO_5_, 482.2901).

Colachalasin K (**2**)

Colorless powder; [*α*]_D_^25^ + 40.2 (*c* 0.2, CH_3_OH); UV (CH_3_OH) *λ*_max_ (log *ε*) 196 (1.23), 215, (0.52), 250 (0.06) nm; ECD (0.25 mm, CH_3_OH) *λ*_max_ (Δ*ε*) 204.5 (5.53), 220 (−2.88), 262.5 (1.14) nm; IR (KBr) *ν*_max_ 2361, 2339, 1702, 1650, 1540, 1457, 1271, 1098 cm^−1^; ^1^H and ^13^C NMR data see Table S2; HRESIMS *m*/*z* 450.2631 [M + H]^+^ (calcd for C_28_H_36_NO_4_, 450.2639).

Colachalasin L (**3**)

Colorless powder; [*α*]_D_^25^ + 25.5 (*c* 0.2, CH_3_OH); UV (CH_3_OH) *λ*_max_ (log *ε*) 195 (1.21), 210 (2.71), 250 (0.26) nm; ECD (0.25 mm, CH_3_OH) *λ*_max_ (Δ*ε*) 211 (−0.53), 263 (1.27) nm; IR (KBr) *ν*_max_ 2807, 2730, 2354, 1660, 1582, 1386, 1350, 1083 cm^−1^; ^1^H and ^13^C NMR data see Table S3; HRESIMS *m*/*z* 464.2430 [M − H]^−^ (calcd for C_28_H_34_NO_5_, 464.2442).

Colachalasin M (**4**)

Colorless oil; [*α*]_D_^25^ + 33.1 (*c* 0.2, CH_3_OH); UV (CH_3_OH) *λ*_max_ (log *ε*) 199 (2.80), 210 (1.63), 220 (0.53) nm; ECD (0.25 mm, CH_3_OH) *λ*_max_ (Δ*ε*) 205 (5.06), 242 (−0.01), 293 (0.43) nm; IR (KBr) *ν*_max_ 2808, 2730, 2354, 2338, 1660, 1386, 1350, 1083 cm^−1^; ^1^H and ^13^C NMR data see Table S3; HRESIMS *m*/*z* 512.2653 [M + H]^+^ (calcd for C_29_H_38_NO_7_, 512.2643).

Avellanin P (**5**)

Yellow oil; [*α*]_D_^25^ + 23.3 (*c* 0.2, CH_3_OH); UV (CH_3_OH) *λ*_max_ (log *ε*) 196 (2.83), 209 (2.73), 245 (0.95) nm; ECD (0.25 mm, CH_3_OH) *λ*_max_ (Δ*ε*) 211 (−7.14), 233 (4.18) nm; IR (KBr) *ν*_max_ 2829, 2708, 1660, 1591, 1381, 1350 cm^−1^; ^1^H and ^13^C NMR data see Table S4; HRESIMS *m*/*z* 562.3037 [M + H]^+^ (calcd for C_31_H_40_N_5_O_5_, 562.3024).

### 3.4. X-Ray Crystallographic Analysis

Colorless crystals of compounds **1**, **6**, **7** were obtained from a mixed solvent of ethanol and H_2_O at room temperature. X-ray crystallographic data were collected using a Bruker APEX-II CCD diffractometer. The crystal structures were solved with the ShelXT structure solution program using Intrinsic Phasing and refined with the ShelXL refinement package through Least Squares minimization. The crystallographic data are provided in Tables S5–S7. The crystallographic data in CIF format have been deposited at the Cambridge Crystallographic Data Centre (deposition number: CCDC 2423674, 2464770, 2464771).

### 3.5. HPLC Analysis of Marfey’s Derivatives

Compound 5 (0.5 mg) was dissolved in 6 N HCl (1 mL) in a sealed glass bottle and incubated at 70 °C for 12h. After cooling, the resultant hydrolysate was dried to remove the remaining HCl, then it was dissolved in 100 μL of H_2_O. Subsequently, 1 M NaHCO_3_ (20 μL) and 1% FDAA (Marfey’s reagent, 100 μL) were added and the mixture, which was then incubated at 40 °C for 1 h. The reaction was quenched by adding 2 N HCl (10 μL). The mixture was dissolved in MeOH (500 μL) and analyzed by HPLC (SilGreen C-18 column, 250 × 4.6 mm) with gradient elution CH_3_CN/H_2_O with 0.1% HCOOH (from 15% to 45% acetonitrile during 55 min, flow rate 1.0 mL/min, detector at 340 nm). The standard amino acids L-Pro, D-Pro, L-*N*MePhe, D-*N*MePhe, L-Ala, D-Ala, L-Leu, D-Leu were treated as the above process. Retention times for the FDAA-derivative amino-acid standards were 28.5 min for L-Pro, 30.5 min for D-Pro, 43.8 min for L-*N*MePhe, 48.8 min for D-*N*MePhe, 43.5 min for L-Ala, 49.0 min for D-Ala, 26.5 min for L-Leu, and 31.5 min for D-Leu. Acid hydrolysate of **5** contained L-Pro (28.5 min), L-*N*MePhe (43.8 min), D-Leu (49.0 min), and L-Ala (26.5 min).

## 4. Conclusions

In conclusion, four new cytochalasins (**1**–**4**), a new cyclic pentapeptides (**5**), and five known compounds (**6**–**10**) were isolated from the endophytic fungus *A. templicola* guided by the analysis of biosynthetic gene clusters and the OSMAC strategy. Their structures were elucidated through extensive spectroscopic, X-ray diffraction analyses, and Marfey’s analysis. In bioactivity assays, compound **2** demonstrated potent anti-inflammatory activity in zebrafish. Compounds **4** and **6** showed cytotoxicity against selected cell lines. These findings provide valuable insights for the development of anti-inflammatory and antitumor agents derived from cytochalasins. These results demonstrate that integrating biosynthetic gene cluster analysis with the OSMAC strategy effectively promotes the production of bioactive secondary metabolites.

## Figures and Tables

**Table 1:** Cytotoxic activities of compounds **1**–**2** and **4**–**7**.

Compound	IC_50_ (μm)				
	K562	ASPC-1	NCI-H446	MDA-MB-231	NCI-H446/EP
**1**	>30	>30	>30	>30	>30
**2**	>30	>30	29.2	>30	>30
**4**	>30	>30	4.2	9.0	6.5
**5**	>30	>30	>30	>30	>30
**6**	>30	>30	2.6	>30	11.2
**7**	>30	>30	>30	>30	>30
DOX ^a^	<1	<1	<1	<1	<1

^a^ DOX (Doxorubicin) was used as positive control.

## Data Availability

Data are contained within the article or Supplementary Materials; further inquiries can be directed to the corresponding author.

## Supplementary Materials

The following supporting information can be downloaded at: https://www.mdpi.com/article/10.3390/md23070285/s1, *HcpA1* Amino acid sequence; crystallographic data of **1**, **6**, and **7**; spectroscopic data including 1D and 2D NMR of **1**–**5**; spectroscopic data including 1D NMR of **6**–**10**; HRESIMS and IR spectra of **1**–**5**; experimental ECD spectra of **1** and **5**; HPLC analysis of Marfey’s derivatives of avellanin P (**5**); Anti-inflammatory Assays [20]; Cytotoxicity Assays [21]. (PDF). CIF file for **1**, **6**, and **7**. (CIF).
