# Supplementary material for: Discovery of Secondary Metabolites from the Sponge-Derived Fungus Aspergillus templicola
Source: Mar Drugs. 2025 Jul 9;23(7):285. doi: 10.3390/md23070285 (PMC12300295; doi:10.3390/md23070285)
Supplement: Supplementary file 1 [file marinedrugs-23-00285-s001.zip › marinedrugs-3745227-supplementary/Supporting Information.pdf]

Supporting Information

Discovery of Secondary Metabolites from the Sponge-Derived Fungus *Aspergillus templicola*

Kai Li [1,2], Yue Zhang [1,2], Lei Li [1,2], Sen Wang [1,2], Cili Wang[1,2,3,*] and Pinglin Li [1,2,*]
[1] Key Laboratory of Marine Drugs, Chinese Ministry of Education, School of Medicine and Pharmacy, Ocean University of China, Qingdao 266003, China
[2] Laboratory for Marine Drugs and Bioproducts, Qingdao Marine Science and Technology Center, Qingdao, 266237, China
[3] Key Laboratory of Marine Food Processing & Safety Control, College of Food Science and Engineering, Ocean University of China, Qing dao 266003, China
**E-mail: wangcili881@163.com; lipinglin@ouc.edu.cn*

Table of Contents

Table S1. Annotation of each protein encoded by the par cluster. 4
Table S2. The ^{1}H (500 MHz) and ^{13}C NMR (125 MHz) data of **1** and **2** in $CDCl_3$ 6
Table S3. The ^{1}H (500 MHz) and ^{13}C NMR (125 MHz) data of **3** and **4** in $CDCl_3$ 7
Table S4. The ^{1}H (500 MHz) and ^{13}C NMR (125 MHz) data of **5** in C_6D_6 8
Table S5. Crystal data and structure refinement for compound **1**..9
Table S6. Crystal data and structure refinement for compound **6**..10
Table S7. Crystal data and structure refinement for compound **7**..11
Figure S1. Structures of colachalasins A, D, F..12
Figure S2. HR-ESI- MS spectra of compound **1**...12
Figure S3. IR spectra of compound **1**..13
Figure S4. The ^{1}H NMR spectrum of compound **1** in $CDCl_3$. 13
Figure S5. The ^{13}C NMR spectrum of compound **1** in $CDCl_3$.......................................14
Figure S6. The HSQC spectrum of compound **1** in $CDCl_3$..14
Figure S7. The HMBC spectrum of compound **1** in $CDCl_3$. 15
Figure S8. The ^{1}H–^{1}H COSY spectrum of compound **1** in $CDCl_3$. 15
Figure S9. The NOESY spectrum of compound **1** in $CDCl_3$...16
Figure S10. HR-ESI-MS spectra of compound **2**..16
Figure S11. IR spectra of compound **2**...17
Figure S12. The ^{1}H NMR spectrum of compound **2** in $CDCl_3$.......................................17
Figure S13. The ^{13}C NMR spectrum of compound **2** in $CDCl_3$......................................18
Figure S14. The HSQC spectrum of compound **2** in $CDCl_3$...18
Figure S15. The HMBC spectrum of compound **2** in $CDCl_3$. 19
Figure S16. The ^{1}H–^{1}H COSY spectrum of compound **2** in $CDCl_3$. 19
Figure S17. The NOESY spectrum of compound **2** in $CDCl_3$..20
Figure S18. HR-ESI-MS spectra of compound **3**..21
Figure S19. IR spectra of compound **3**...21
Figure S20. The ^{1}H NMR spectrum of compound **3** in $CDCl_3$.......................................22
Figure S21. The ^{13}C NMR spectrum of compound **3** in $CDCl_3$......................................22
Figure S22. The HSQC spectrum of compound **3** in $CDCl_3$...23
Figure S23. The HMBC spectrum of compound **3** in $CDCl_3$. 23
Figure S24. The ^{1}H–^{1}H COSY spectrum of compound **3** in $CDCl_3$. 24
Figure S25. The NOESY spectrum of compound **3** in $CDCl_3$..24
Figure S26. HR-ESI-MS spectra of compound **4**..25
Figure S27. IR spectra of compound **4**...25
Figure S28. The ^{1}H NMR spectrum of compound **4** in $CDCl_3$.......................................26
Figure S29. The ^{13}C NMR spectrum of compound **4** in $CDCl_3$......................................26
Figure S30. The HSQC spectrum of compound **4** in $CDCl_3$...27
Figure S31. The HMBC spectrum of compound **4** in $CDCl_3$. 27
Figure S32. The ^{1}H–^{1}H COSY spectrum of compound **4** in $CDCl_3$. 28
Figure S33. The NOESY spectrum of compound **4** in $CDCl_3$..28
Figure S34. HR-ESI-MS spectra of compound **5**..29
Figure S35. IR spectra of compound **5**...29

Figure S36. The ^{1}H NMR spectrum of compound **5** in C_6D_6. 30
Figure S37. The ^{13}C NMR spectrum of compound **5** in C_6D_6. 30
Figure S38. The HSQC spectrum of compound **5** in C_6D_6. 31
Figure S39. The HMBC spectrum of compound **5** in C_6D_6. 31
Figure S40. The ^{1}H–^{1}H COSY spectrum of compound **5** in C_6D_6. 32
Figure S41. The ^{1}H NMR spectrum of compound **6** in $CDCl_3$. 32
Figure S42. The ^{13}C NMR spectrum of compound **6** in $CDCl_3$. 33
Figure S43. The ^{1}H NMR spectrum of compound **7** in $CDCl_3$. 33
Figure S44. The ^{1}H NMR spectrum of compound **8** in $CDCl_3$. 34
Figure S45. The ^{13}C NMR spectrum of compound **8** in $CDCl_3$. 34
Figure S46. The ^{1}H NMR spectrum of compound **9** in $CDCl_3$. 35
Figure S47. The ^{13}C NMR spectrum of compound **9** in $CDCl_3$. 35
Figure S48. The ^{1}H NMR spectrum of compound **10** in CD_3OD. 36
Figure S49. The ^{13}C NMR spectrum of compound **10** in CD_3OD. 36
Figure S50. Experimental ECD spectra of **1** and **5**. 37
Figure S51. HPLC analysis of Marfey's derivatives of avellanin P (**5**). 37
Anti-inflammatory Assays. 38
Cytotoxicity Assays. 38

Table S1. Annotation of each protein encoded by the par cluster.

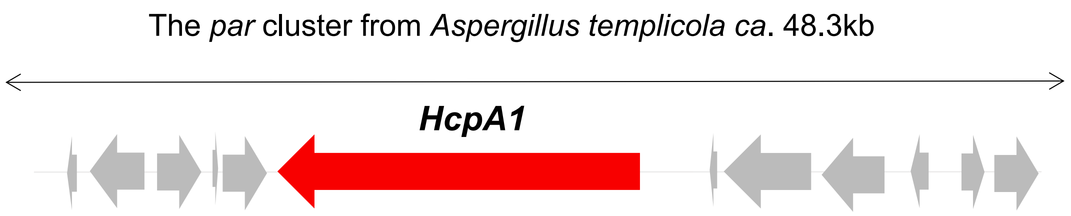

Gene	Amino acids(base pairs)	Protein homologue, origin	Similarity/Identity	Proposed function
hcpA1	5271(15813)	*hcpA*, *Penicillium chrysogenum*	73/57	Scaffold biosynthesis

Amino acid sequence

HcpA1

*MAQPVPCRLPKFSPSSVDGPKRPVSMRVKTSSSQTTQLLSSWEQGQLSSLLQAAWALLLYRYTG
CEDVCFGYQHLGAEDIQQASQDTATKSSIMHLSVKEHESVQQVLKKVGRKGHCTDTLEKRPR
KTGDHGDYGVFNTTLTVRVCTGSTRRARDAFVQPALAMTLPEECRARLLVKILEQDVGIFVEW
WNQDMSVEQAKSIANYFEQTLACLLSAEEVAVKVLNIISEQDWRRICQFNSVPPASPNRCIHEVI
YEQTLLRPEREAVCSWDGSLTYRDLDLQASKVAAYLLQHGVKPETNVAVCFDKSKWYIVAVLG
VLKAGGAFVPLDPTHPESRLQSLVRSVQAQTMLCSRDRVDKLSSVAEHIVPLDDEALIEFPPAV
DTSVLSPEVRGSNAAYVIFTSGSTGEPKGTLMEHKSYVASSVAHAPRLRIDSESRLLQFAAHTFD
ASLVDILTVLMQGACICIPSEEERLNDIVKAINNTRVNHASLTPSFIDFIDIADVPGLETLALVGEA
MSQSHIETWSKINLLNGFGPTEAAVTAAINSNVTLSSDSRDIGFPTGAHCWIVDPEDHDRLVPV
GCVGEMLLEGPTLARGYLNNPEKTAESFIYDPAWTKYDGSASPGRKDTQIKLHGQRVELGEIE
DNLNSDSDIKHCLVLLPKTGFAEGRLVAVLSLSVLADDDNNADVVPLKLVEGQQKSQLISDIRE
RLSARLPAYMIPAVWLCVEALPVLASRKLDRKATASWVAEMENDPEVRSHQTIDGNDATFNRSA
NPMEDHIATIWSRILNIPKHNIPLGESFLSLGGDSIAAITCMGFCKKKGIGITVQEILRSKSIKELA
TRAKEIEQPAAYEEVIGKPFDLSPIQKLHFMVRREGQGHFNQSVLTRLNGKIGLEDLRRSLEILI
DRHSMLRARLIDPGPEAEFQQVVTSNVKGSYRWRVHEETCAEAIERSVAESQSSINAFVGPVIAV
DTFCEDGQISVLSIVAHHLVVDIVSWRIILEDLEDLLLNGPGTTSQNDSLPFQTWCDLQAERCRI
LPKEGIKLPAPELDYWGMESHPITYGEVDCEGFEVDLQSTTCLLMDCHNSLQTEPIDIFLAALL
HSFRQTFTDRPLPIIYNEGHGREVWDSSIDVSRTVGWFTILYPILLAEPVSDDIVETVVRVKDLRR
RVSDNGRQEFAQRMCIGEGHRKSTHASPMEMSFNYVGQHRDLQRQDGLFQLMNQMAGETG
RGGGAADFGEDTPRFALFEISAMAAHGKLRFTFSFNKYMDHQLRIRNWISCCHDTLKQVGPK
LQSLTPRPTLSTFPMLSLTYKDLEDVLPQKLRSIGVESFAMVEDIYPCTPMQEGILLSRTRDQSL
YAVHNTFEVQVSGRKPDIDRLAAAWKAVVSCHSLLRTVFVQGLTKRDLFSQVVLKSSDSNLDVV
NCSSDDDVLSVLEGWESIHADHNRPPNQFTICATATDKVFCRLELSHACMDGFSISIILQDLQR
AYHGSLDKDRTPMFKNYVQHLLESSHTASMEYWKDYLSDPKPSHFPILNDGKELTKQLRTMH
LSFDLYDTLQKACEENGFTLSTAFSAAWGLTIRSFCNTDDVCFLYMASLRDLPVEDLETVVGPV
INLLTCRMKVSDDEPLIDVLLRMQQDYMEQVPHRQTPLIDIQRALKPSDTTLCNTGLSYRRLPP
TNQNADGEIQFLELGSIHDPAEFPIFVNVEVSDRGANVDLNYWTTALSDRQAGSVAKTFIKHLE
TITLHQDTQMRLVNGLSDWHLQQIMTWNKKLPETVEKCAHEVLSQKAKSNPDQYAVSAWDGE*

LTYSRLDELSSALSAYLMQLGVGQGSLVPMELPRSVWRVVAIAAVLKSGGVCIPVIEGRLQETLN
DDLITDEVQVALASPTRAHLMEGTIPYVVPINDSLFNYLPRSKEQFVSFSQPEDDAYVVYSFDEK
STPKIVMLDHQTIMTRSQNFASKVGLNTRTRLFQLSDYTSDMFLQEVVGAWSQGACICIPSDSE
SQHPSLSINALHANMISTTPQTASDMQPLDVPDVQTLVVHAKHVDLKLKTTWLGKVQLHTFYG
KAEYSSTCVHASYSDAHIGAVPTGSGVGCRLWLVDPQDHERLVPIGCPGELIVEGPVIADHYRH
TESLASQESIERPKWALSIQHETEDDGTEFRKMFKTGDLARYNSDGTLAYIAEQDARNQNQEAT
AKISSADWWKSYLVDAEACLFPSLQYQKRQRDLVSSAFPAKNVAMIRASCQKLGVDVGSLLRVA
WGLVLRCYTGSEEVCFGHRHMGQDITPIRLHLKDDMALEEVVCKLESDVLQILQHPSSLLTIQE
ECKFESMMFNTFFDYSDMLDDGGQDQASFKPLSDIAMSDYIIGVSAQVSNSFITLSLICATDCLS
QANIADILECYQLVLDSTVNAIQQNRCIGDVEFLTETSRKQIQKWNAALPDRPTRCANEIIEEQV
YRMPPSTPAICSWDGEFTYEDLDFFSTRLANYLREIGVKPETFVPLCFEKSAWAIISQIAVLKAG
GAFVSLDPAHPEERMKNMIQDLDAKIILCSAKYHQKASGVCDLALSVYSTAVMELPSPTPASQT
AIPTINNPAYAIFTSGTTGKPKATVIEHVGLCSISLSVGNTYEFGPGTRCMQFSSYTWDVSILESIII
LMNGGCVCVPSEEERMNDLAGAIRRMEANLLIGSPSVANTLDPKAVPSLRTILAGGEKTTASHV
ERWSDRCVINAYGPSEATIMATSCMIVGKDGTRHTMDYSSIGTPHGGRAWVVDPHNYNRLLPV
GAVGELVLEGCNVARGYLNNEQKTKEAFIHDPHWARHHGLRDAFKRQERMYRTGDLVHYNE
DGSLSFVARKDTQIKLHGQRVELGEIEQQCIRFLPPGTEVAVDLVTPEVKSVASCLAVFFTIDKH
QVQEGSSLHDSNSEILVSMAGNRDETLKQQLIVFLKHALPSAFIPKLFVPVKHLPLTTAGKLDR
RRLKSLVESLSREKLRSYGVSNAREGRILNKGLATTLMALWEEAIGLEPESIAAGDNFFEIGGDSY
SAMKLVSAAGSCGISLKVTDIYAHPILADLATQCKPREASAENRTVEPFSLLPQFVDRDEILQEV
ADKCSVQKESIFDIYPCSPAQEGLLTLSLKQQGAYIAQPVFRLSNKIDLQRFKEAWQQVVDDLD
ILRTRIVHTDSTNFLQAVLKTSILDPSAVPSLKVLVCGGEKLSKEVVTKWAHRVQLVNGYGPTET
TIFAVMNDVSANPEPSCIGYGIPCTLTWIVDPENHDRLFPLGAVGELVLEGPALAREYLKNPEK
TAAAFVNNPAWMKRFPSAQKSSRRIYKTGDLVKYNPDGSIECIGRKDHQVKLHGQRMELGEIE
HRLYEDFRVRHAVVILPRSGPFRDRLVTILSLDSLTLDKSMISDGTCELVSRDLMGPAYSELTEIQ
KGLESQLPIYMVPQTWAVLKTLPMLVSGKLDRKRIANWIDNAGEEIHDRIMQDYDIIKRGNFEE
KKETNDGSVVETLRDIFVQILNIPSEKVDTNRSFVSLGGDSITGMAVVSRARKHGLSLTLHSVLQ
SKSIKELALAAGTKVKTIQVKERHEELFDLSPIQRLYFDSASDFGGASRFNQGMTVRLNRKIPAE
TVKRAVEAVVQRHSMLRARFVKSRDGKWQQRIGADVGSSYRFRTHTVTDKHEMRFRLAESQN
LLDIHSGPICAGDLFELQGHGQILALIVHHLCVDMVSWRIILQDIQDCIELGSLSPDKTLSFQSW
CELQTREAREGSLFQLPLQAQQPNLEYWGMEKARNLYGHVKMETFTVTQEATSFILGSCHDV
FGTETLEVLLAAVIHSFNQVFTDRDAPTIYNEGHGREPWDDSIDLSRTVGWFTTFCPLHVGKSK
DLFDTLRYVKDVRRAIFGNSRSYFSQDLLRTRKEGGVTQFPVPLEMVFNYLGQLQQLERDDSL
FQHYDDGSSFEALDSASDMGPETPRFALFELSAIIIKERLHMSFTYNRQTKHESRIKRWASECKK
ALEEGIPRLQDWLPEPTLSDYPLLSITYDGLKNMMRDILPKAGIESWESVEDIYPCSPVQEGILLS
QLRDPHGYMFHAIIEIRHSDGSSRVEANKVRKAWSMVVERHPILRTMFIDSYCKGGSFDQLVLK
AVEDEIVEFECDERIAFETLDNIKLANINAKRLRKLHQQLTICTTTSGRVFLKFEINHAIIDGASV
DLLLRDLQMAYDGQLPPGTGPLFSEYIKHTKATDHNKSLTHWVEYLSNVRPCHLSISSDARVER
RLGSVMMNFDRFSELQKFCEGNSVTLANVTLSAWAIVLRSFTGINDVCFGYLSAGRDSPVPGIQ
DAVGIFINMLCCRVKFEPSQKLCDISRRVQDDFFKALPYQNCSLAQIQHEIGREGQMLFNTALS
IQNHAVSENKKESTISFEFQRAHDPTEYPVTVNVETTKGREGILIRYWADAVSERQAQSLADAIA
QIFTSFIERPSASISELNIHGQSPDVALRDSNSTKRVSLDEEYLQALVDKRIKEALYQMLHEGMLN
VPVLTEKNVNETNTLHFKVPHDYHQTVLTHNMTTSDSMPTLADVRGTSDLENQLWRLWSAAL
GLSSDNVIHHASFFKLGGDSITAMKMVGAAREEGLMITVADVFNNPVFDDMLATICSRGSTPSC

TPDISSDSTLDRNTESPVVISRSPSPVDITAVKPIQLNEASVQNDIGSKIGLFRGGVADILPVTDFQ ALSLTATLFKSRWMLNYFYLDGKGALDTTRLTESFLQVTNAFDILRTVFVCFHGQCFQVILRKM RPEIFIHDTEGSLDEYTASLQQRDRAHEPRQGEQFVQFYIVRQKGTDRHRILIRMSHAQYDGVC LPRIMSAIKMGYEGLPIPPAPSFANYMRMLPGTVTPEHYQHWKKLLHNSTMTEVIRRNSPNSFQ HIGAFTEQNRTIEIPATALDNVTIATVMQSAWALTLAKLSSQSDVIFGLATSGRNTTIPGIENVVG PCLNTLPVRVKFGDRWTGLDLFRYLQDQQVANMAYESLGFREIIRQCTNWPESTYFTTTVFHQ SLEYEGEMQLDDHDYKMGGVGVIDNFVDLTLFSKQTSTHHLLISLGYSLKGPIQSSYATKVLDM VCETVQSLVANPSIALPSPRTLRSLPSQVVPDLPRQSDEHFLSSHLNTRSISEVLVHSDMVSRVWE QVLPNKNPELPRPPFQLNSSFFELGGDVFNMAQVVWLLEQEGIRVHLEDLLDHPSFLGHMAV LALQNTKQKSLPHAPKLDIRLPPTPPPRPASPATKGGWKPLGKAVTLARKISKWNLASRSRTSLA DVES

Table S2. The ^{1}H (500 MHz) and ^{13}C NMR (125 MHz) data of **1** and **2** in $CDCl_3$

No.	**1**		**2**	
	δ$_C$, type	δ$_H$, muti. (J in Hz)	δ$_C$, type	δ$_H$, muti. (J in Hz)
1	176.0, C		173.1, C	
3	55.7, CH	3.14, m	54.2, CH	3.67, t (7.1)
4	54.5, CH	2.37, t (4.6)	49.2, CH	3.19, d (6.0)
5	34.2, CH	2.73[α]	35.9, CH	2.29, m
6	140.4, C		57.2, C	
7	124.4, CH	5.42, brd (3.0)	61.2, CH	2.75, d (5.4）
8	49.4, CH	2.73[α]	46.5, CH	3.01, dd (10.0, 5.4）
9	80.2, C		85.1, C	
10	45.3, CH_2	2.62, dd (13.7, 10.4); 3.04, dd (13.6, 3.3)	45.6, CH_2	2.79, d (7.2)
11	14.9, CH_3	1.29, d (7.4)	12.8, CH_3	0.99, d (5.3）
12	20.3, CH_3	1.80, s	19.6, CH_3	1.17, s
13	130.3, CH	5.65, dd (15.2, 9.5)	123.9, CH	6.05, dd (15.5, 10.0)
14	131.8, CH	5.55, m	139.2, CH	5.18, ddd (15.5, 11.1, 3.6)
15	37.6, CH_2	2.21, m; 2.12, m	44.4, CH_2	2.11, m; 1.93, m
16	34.9, CH	1.80, m	27.4, CH	1.38, m
17	76.0, CH	3.42, dd (7.9, 3.5)	44.8, CH_2	1.82, m; 1.62, m
18	40.6, CH	2.51, m	34.5, CH	2.61, m
19	152.8, CH	7.04, dd (15.8, 8.3)	161.9, CH	7.21, dd (15.9, 4.2)
20	121.2, CH	5.89, d (15.8)	118.9, CH	5.56, d (15.9)
21	167.4, C		167.4, C	
22	57.6, CH_3	3.74, s	27.5, CH_3	0.97, d (5.4)
23	13.2, CH_3	0.92, d (6.8)	14.1, CH_3	1.05, d (6.7)
24	16.7, CH_3	1.02, d (6.8)		
1’	137.7, C		137.1, C	
2’/6’	129.2, CH	7.21, m	129.4, CH	7.16, m
3’/5’	129.0, CH	7.34, m	129.1, CH	7.34, t (7.5)
4’	127.3, CH	7.28, m	127.2, CH	7.26, m

[a] Recorded at 125 MHz. [b] Recorded at 500 MHz. [α] Overlapped signals

Table S3. The ^{1}H (500 MHz) and ^{13}C NMR (125 MHz) data of **3** and **4** in $CDCl_3$

No.	**3**		**4**	
	δ_C, type	δ_H, muti. (*J* in Hz)	δ_C, type	δ_H, muti. (*J* in Hz)
1	171.9, C		171.2	
3	59.9, CH	3.46, t (7.5)	53.7, CH	3.31, m
4	50.7, CH	3.30, brd	49.3, CH	3.05, dd (5.6, 2.2)
5	125.6, C		31.6, CH	3.25, m
6	133.5, C		148.9, C	
7	69.9, CH	4.06, d (10.1)	70.2, CH	3.93, d (10.4)
8	51.5, CH	3.17, d (10.2)	49.7, CH	3.35, t (10.2)
9	83.1, C		84.5, C	
10	43.5, CH_2	2.87, dd (13.2, 6.4); 2.95, dd (13.2, 8.3)	44.5, CH_2	2.8, dd (7.1, 3.9)
11	17.8, CH_3	1.54, s	13.6, CH_3	1.02, d (6.7)
12	14.2, CH_3	1.70, s	115.3, CH_2	5.27, s
13	128.5, CH	6.33, dd (15.4, 10.3)	126.8, CH	5.97, dd （15.3, 9.9）
14	137.1, CH	5.61, dt (14.8, 7.0)	137.3, CH	5.35, m
15	37.7, CH_2	2.36, m	39.4, CH_2	2.21, m
16	44.1, CH	2.73, m	40.6, CH	2.92, m
17	216.8, C		212.6, C	
18	42.3, CH	3.22, m	79.5, C	
19	29.3, CH_2	1.59, m;2.06, m	149.6, CH	6.74, d (15.8)
20	33.2, CH_2	2.43, m	124.4, CH	6.03, d (15.8)
21	172.4, C		166.3, C	
22	18.7, CH_3	1.20, d (6.4)	20.6, CH_3	1.18, d (6.9)
23	16.2, CH_3	1.13, s	20.7, CH_3	1.59, s
24			53.6, CH_3	5.3, s
1’	137.4, C		137.4, C	
2’/6’	129.3, CH	7.16, d (7.1)	129.4, CH	7.15, m
3’/5’	129.1, CH	7,34, t (7.4)	129.1, CH	7.31, m
4’	127.2, CH	7.27, m	127.2, CH	7.25, m

[a] Recorded at 125 MHz. [b] Recorded at 500 MHz. [α] Overlapped signals

Table S4. The ^{1}H (500 MHz) and ^{13}C NMR (125 MHz) data of **5** in C_6D_6

Pos.	5	
	δ_C[a] type	δ_H[b] muti. (*J* in Hz)
	Ant	
1	169, C	
2	127.1, C	
3	125.5, CH	6.85, m
4	123.2, CH	6.76, m
5	130.9, CH	7.03, m
6	122.9, CH	8.57, d
7	136, C	
NH		9.30, s
	Pro	
1	172.2, C	
2	57.4, CH	3.93, m
3	28.2, CH_2	1.17, m
4	24.4, CH_2	1.43, m; 1.03, m
5	50.0, CH_2	2.70, m
	*N*MePhe	
1	168.7, C	
2	69.4, CH	3.47, m
3	34.9, CH_2	3.67, dd (13.9, 11.6);<br>3.83, dd (13.9, 4.7)
4	139.2, C	
5,9	129.6, CH	6.91, m
6,8	126.9, CH	7.02[α]
7	128.5, CH	7.02[α]
*N*Me	39.6, CH_3	2.01, s
	Leu	
1	171.7, C	
2	52.9, CH	5.10, m
3	40.2, CH_2	2.41, m; 2.01, m
4	25.9, CH	1.89, m
5	23.5, CH_3	0.93, d (6.7)
6	21.4, CH_3	0.97, d (6.7)
NH		7.01[α]
	Ala	
1	171.2, C	
2	50.3, CH	5.19, m
3	17.8, CH_3	1.52, d (7.3)
NH		7.54, d (9.1)

[a] Recorded at 125 MHz. [b] Recorded at 500 MHz. [α] Overlapped signals

Table S5. Crystal data and structure refinement for compound **1**.

Identification code	cu_20241219A_0m
Empirical formula	$C_{31}H_{47}NO_7$
Formula weight	545.69
Temperature/K	300.00
Crystal system	orthorhombic
Space group	$P2_12_12_1$
a/Å	6.9307(11)
b/Å	14.161(2)
c/Å	32.047(3)
α/°	90
β/°	90
γ/°	90
Volume/Å^3	3145.3(8)
Z	4
ρ_{calc}g/cm^3	1.152
μ/mm^{-1}	0.651
F(000)	1184.0
Crystal size/mm^3	0.16 × 0.08 × 0.08
Radiation	Cu Kα (λ = 1.54178)
2Θ range for data collection/°	6.824 to 149.576
Index ranges	$-8 \leq h \leq 8$, $-17 \leq k \leq 17$, $-38 \leq l \leq 40$
Reflections collected	46514
Independent reflections	6449 [R_{int} = 0.0963, R_{sigma} = 0.0408]
Data/restraints/parameters	6449/35/363
Goodness-of-fit on F^2	1.058
Final R indexes [I>=2σ (I)]	R_1 = 0.0567, wR_2 = 0.1618
Final R indexes [all data]	R_1 = 0.0693, wR_2 = 0.1762
Largest diff. peak/hole / e Å^{-3}	0.67/-0.36
Flack parameter	0.03(11)

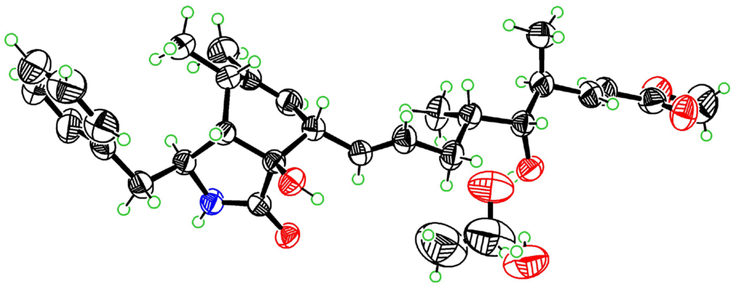

X-ray crystal structure of **1**.

The ellipsoids of non-hydrogen atoms of **1** are shown at 50% probability levels.

Table S6. Crystal data and structure refinement for compound **6**.

Identification code	cu_0222_6_0m
Empirical formula	$C_{28}H_{35}NO_6$
Formula weight	481.57
Temperature/K	150.00
Crystal system	monoclinic
Space group	$P2_1$
a/Å	10.0138(8)
b/Å	11.7363(9)
c/Å	11.1390(8)
α/°	90
β/°	100.345(3)
γ/°	90
Volume/Å^3	1287.83(17)
Z	2
ρ_{calc}g/cm^3	1.242
μ/mm^{-1}	0.705
F(000)	516.0
Crystal size/mm^3	0.2 × 0.15 × 0.1
Radiation	CuKα (λ = 1.54178)
2Θ range for data collection/°	8.976 to 150.086
Index ranges	$-12 \leq h \leq 12$, $-14 \leq k \leq 14$, $-13 \leq l \leq 13$
Reflections collected	12349
Independent reflections	5043 [R_{int} = 0.0230, R_{sigma} = 0.0300]
Data/restraints/parameters	5043/3/322
Goodness-of-fit on F^2	1.090
Final R indexes [I>=2σ (I)]	R_1 = 0.0737, wR_2 = 0.1766
Final R indexes [all data]	R_1 = 0.0743, wR_2 = 0.1778
Largest diff. peak/hole / e Å^{-3}	0.58/-0.27
Flack parameter	0.06(6)

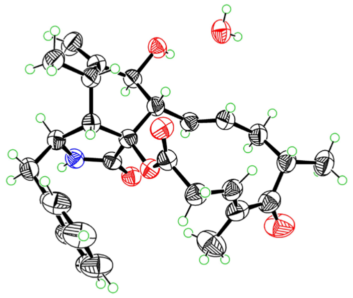

X-ray crystal structure of **6**.

The ellipsoids of non-hydrogen atoms of **6** are shown at 50% probability levels.

Table S7. Crystal data and structure refinement for compound **7**.

Identification code	cu_0222_5_0m
Empirical formula	$C_{28}H_{35}NO_4$
Formula weight	449.57
Temperature/K	150.00
Crystal system	trigonal
Space group	$P3_121$
a/Å	11.995(8)
b/Å	11.995(8)
c/Å	30.450(14)
α/°	90
β/°	90
γ/°	120
Volume/Å^3	3794(5)
Z	6
ρ_{calc}g/cm^3	1.181
μ/mm^{-1}	0.621
F(000)	1452.0
Crystal size/mm^3	0.2 × 0.15 × 0.1
Radiation	CuKα (λ = 1.54178)
2Θ range for data collection/°	8.512 to 149.278
Index ranges	$-14 \leq h \leq 13$, $-14 \leq k \leq 14$, $-37 \leq l \leq 37$
Reflections collected	20003
Independent reflections	5135 [R_{int} = 0.0420, R_{sigma} = 0.0351]
Data/restraints/parameters	5135/0/343
Goodness-of-fit on F^2	1.036
Final R indexes [I>=2σ (I)]	R_1 = 0.0461, wR_2 = 0.1279
Final R indexes [all data]	R_1 = 0.0658, wR_2 = 0.1443
Largest diff. peak/hole / e Å^{-3}	0.11/-0.08
Flack parameter	0.18(19)

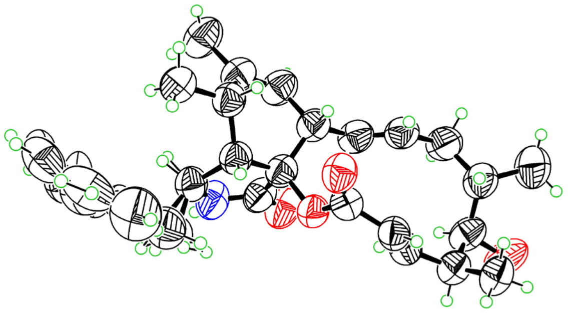

X-ray crystal structure of **7**.

The ellipsoids of non-hydrogen atoms of **7** are shown at 50% probability levels.

Figure S1. Structures of colachalasins A, D, F.

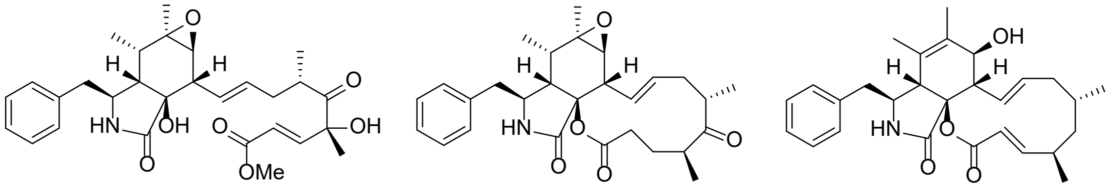

Figure S2. HR-ESI- MS spectra of compound **1**.

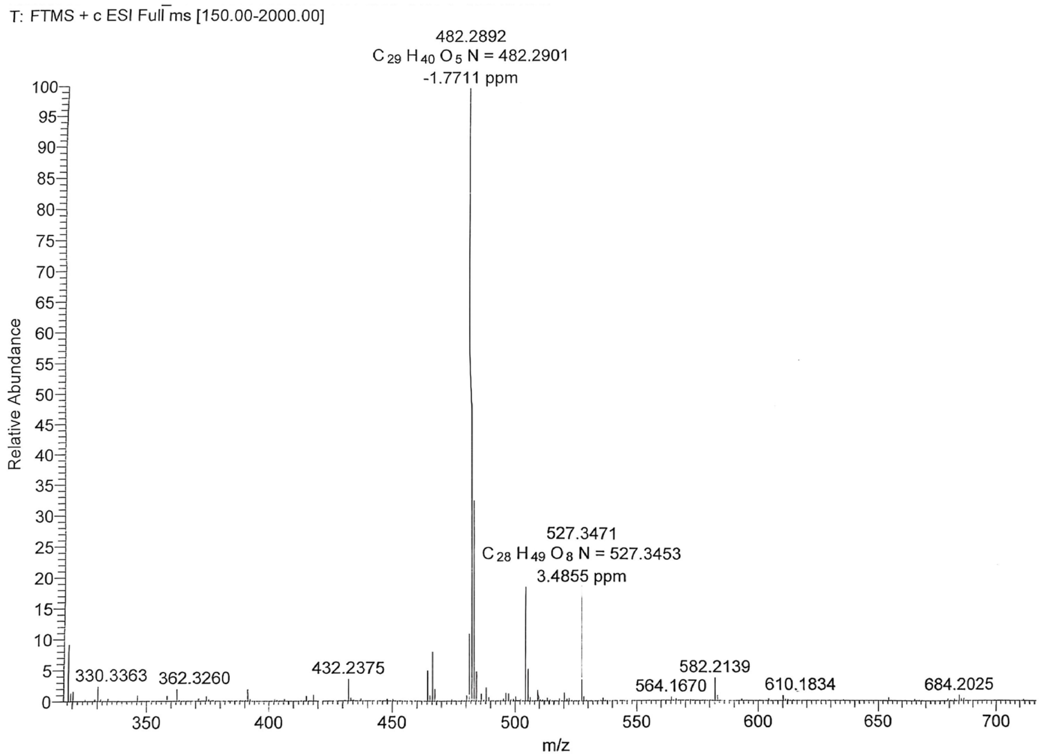

Figure S3. IR spectra of compound **1**.

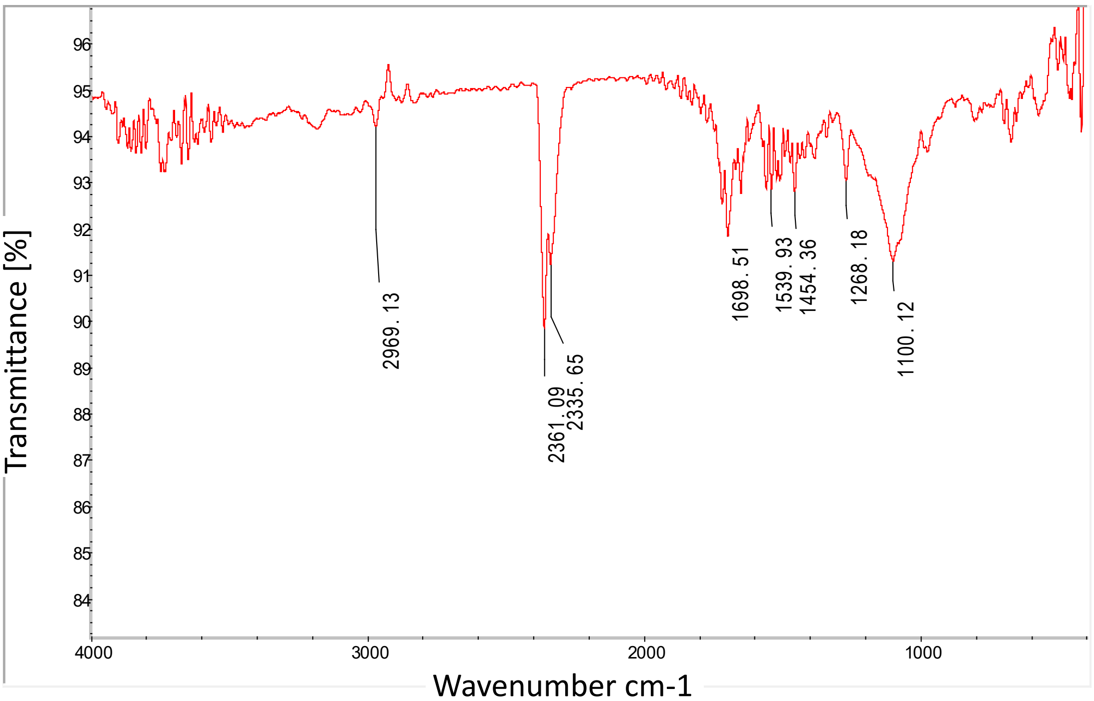

Figure S4. The ^{1}H NMR spectrum of compound **1** in $CDCl_3$.

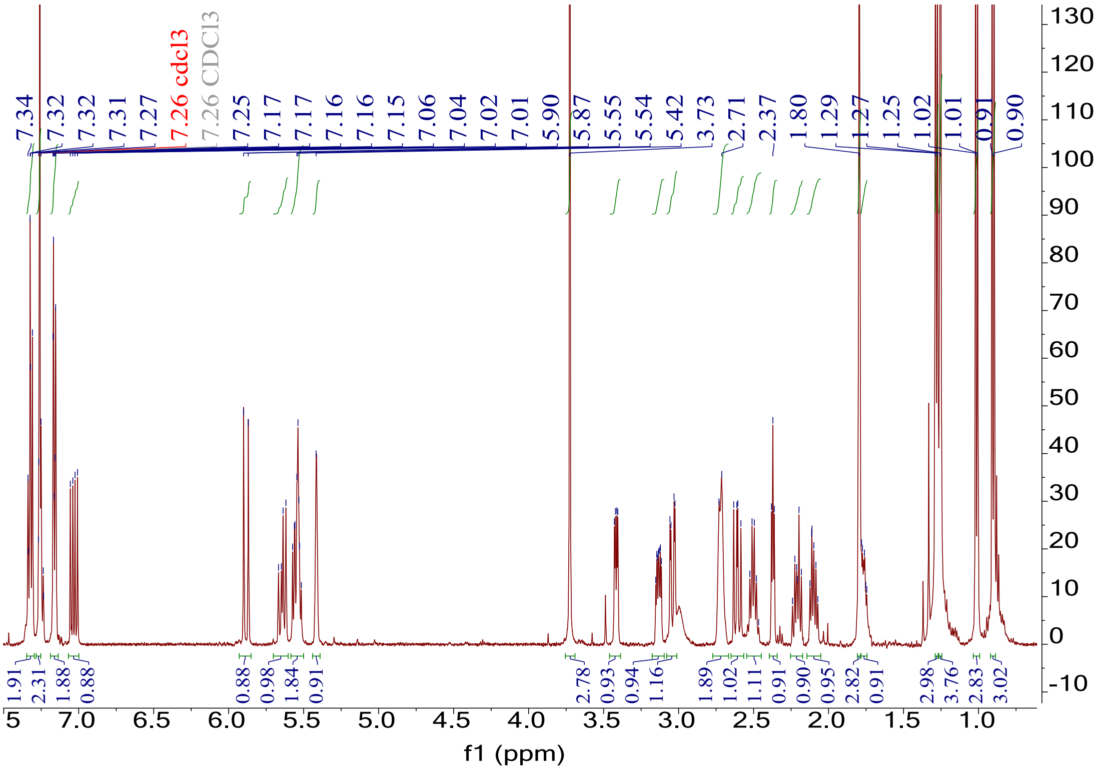

Figure S5. The ^{13}C NMR spectrum of compound **1** in $CDCl_3$.

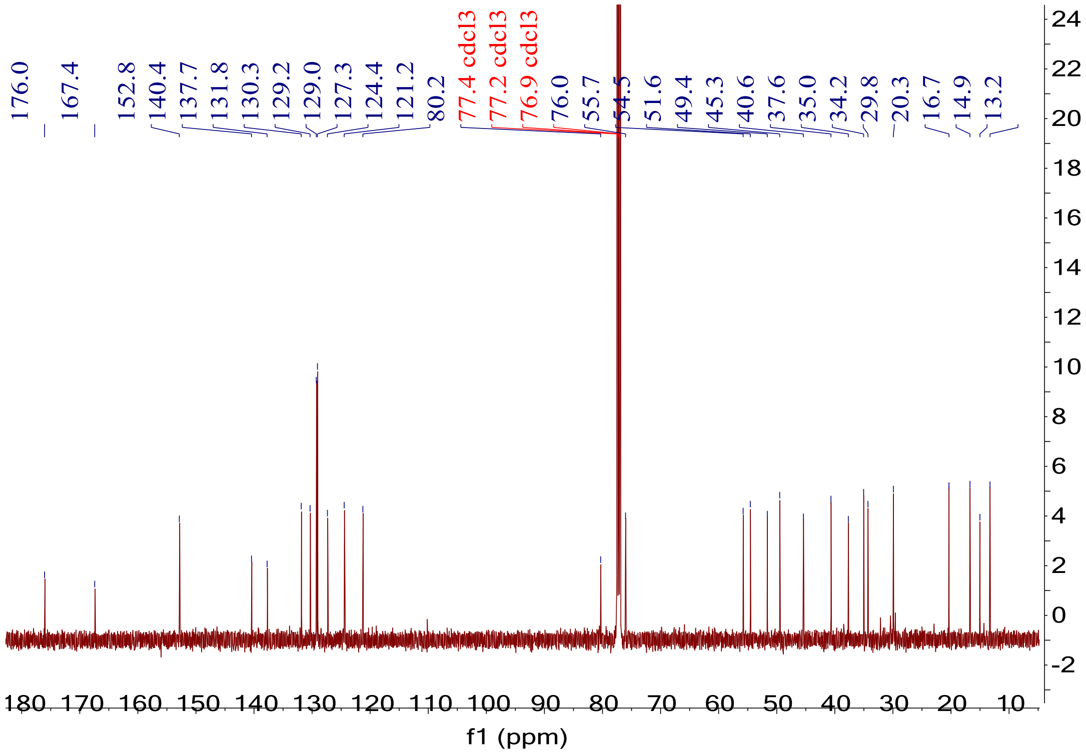

Figure S6. The HSQC spectrum of compound **1** in $CDCl_3$.

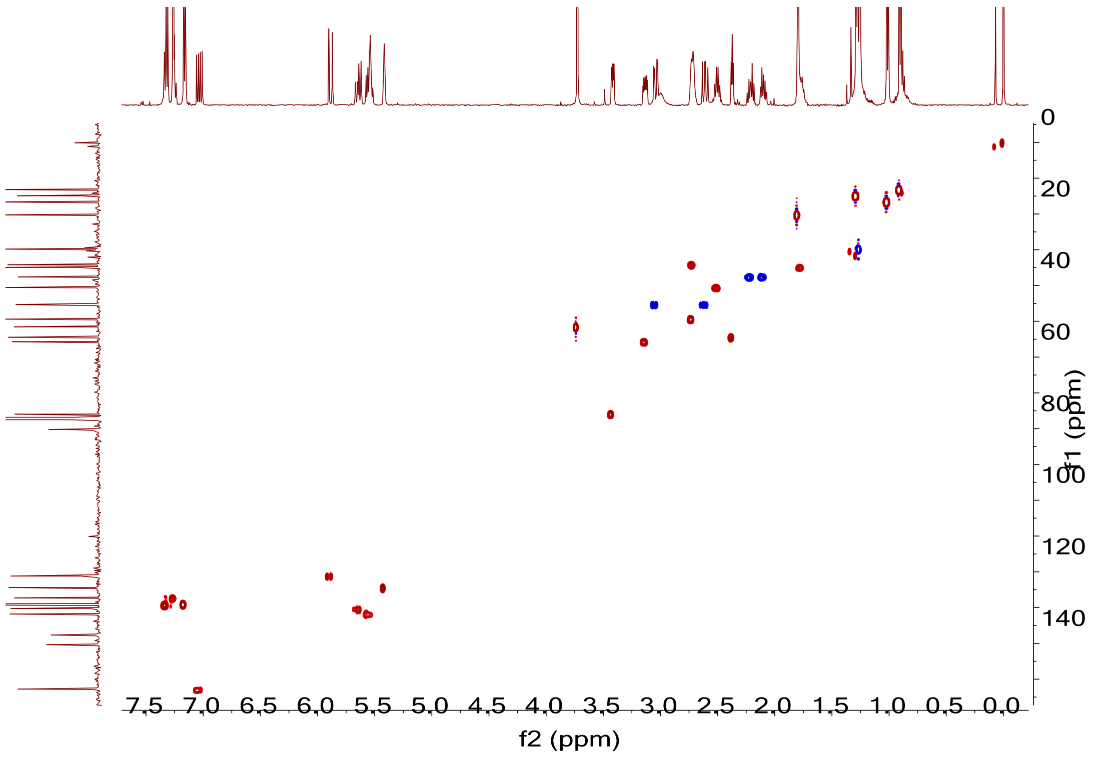

Figure S7. The HMBC spectrum of compound **1** in $CDCl_3$.

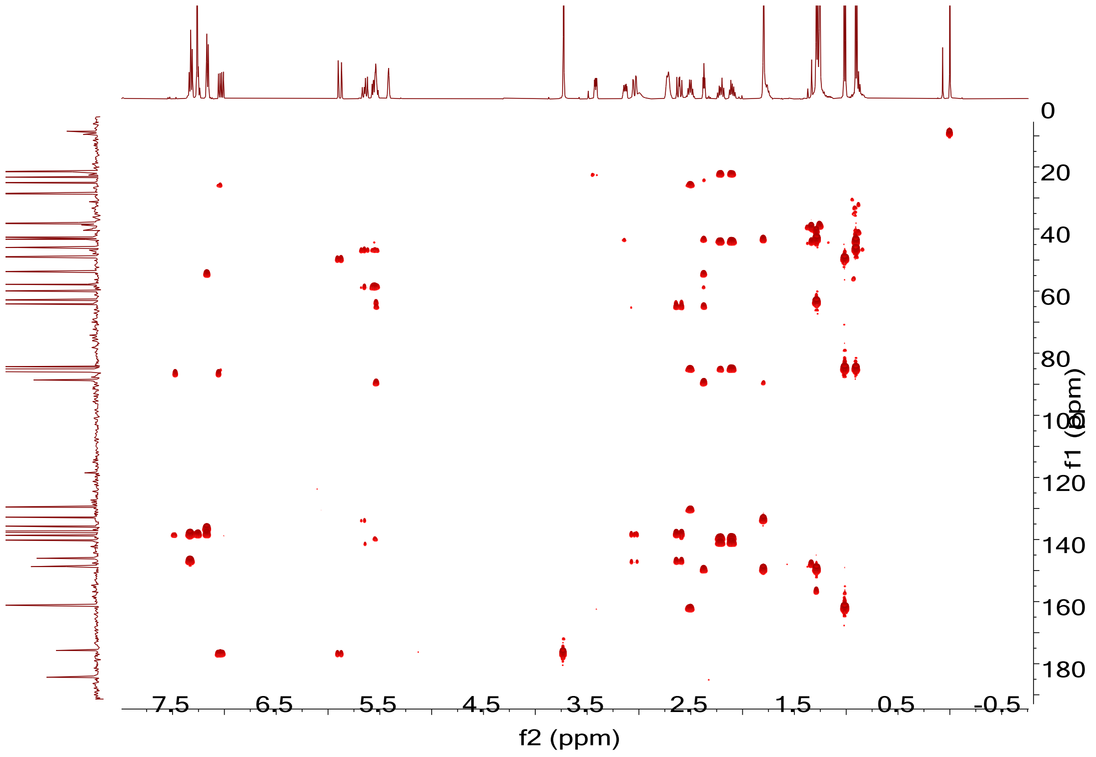

Figure S8. The ^1H–^1H COSY spectrum of compound **1** in $CDCl_3$.

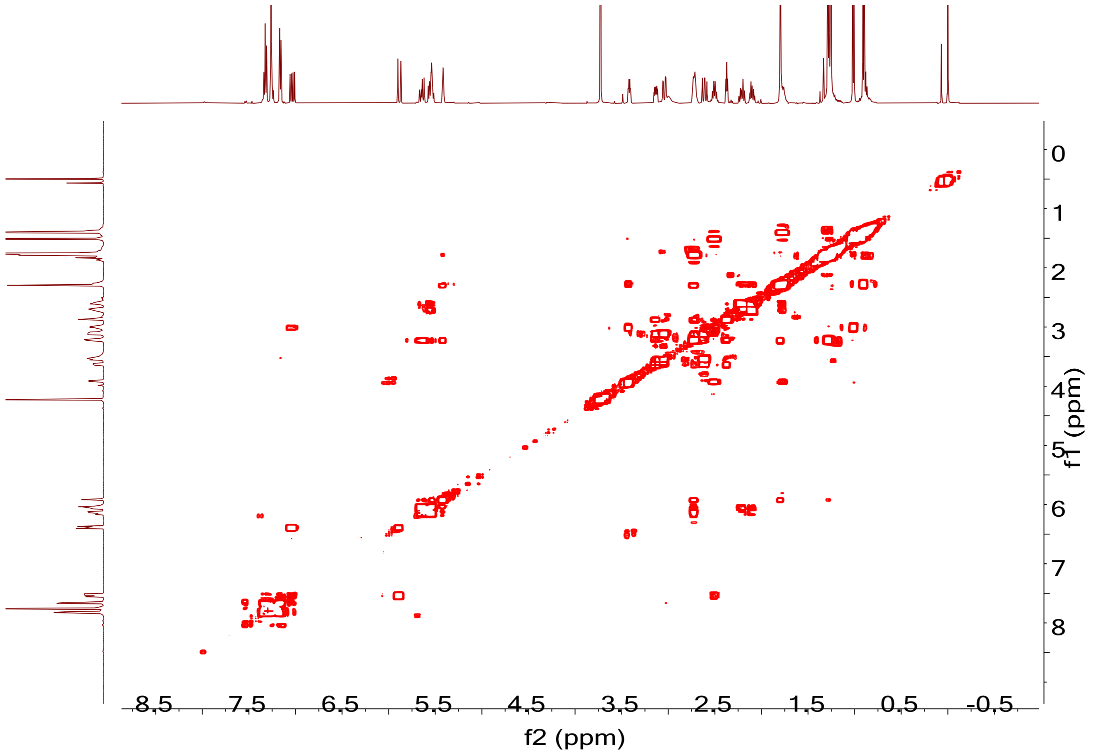

Figure S9. The NOESY spectrum of compound **1** in $CDCl_3$.

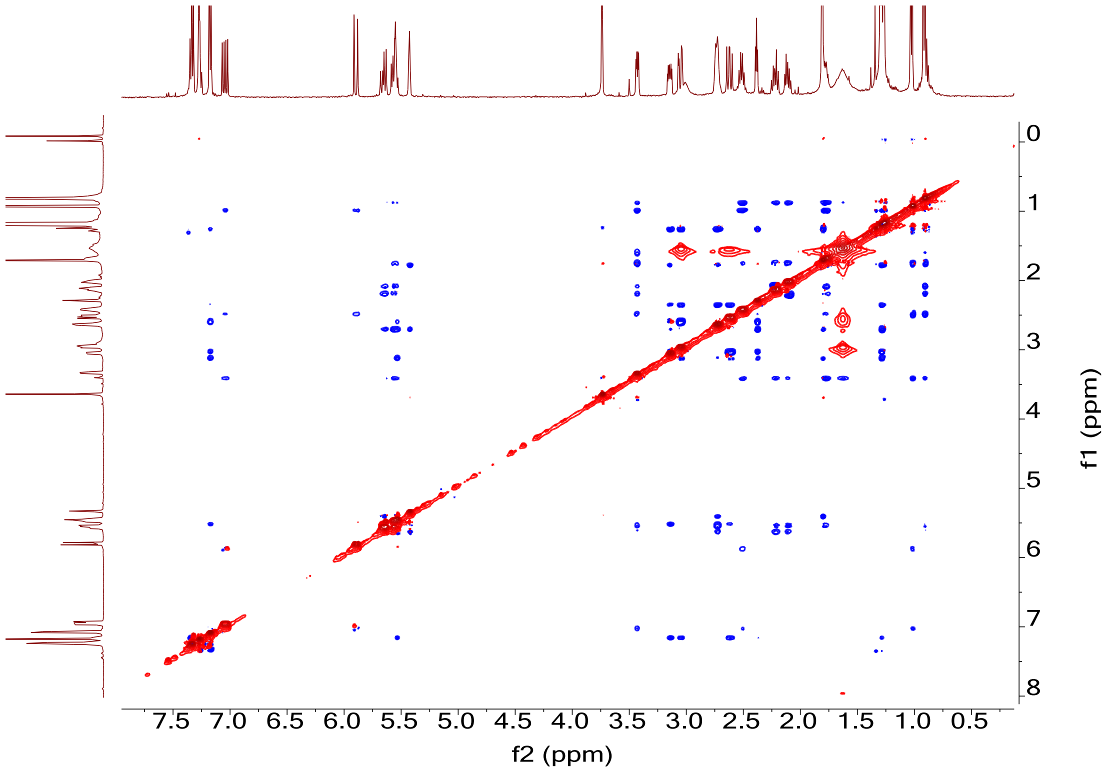

Figure S10. HR-ESI-MS spectra of compound **2**.

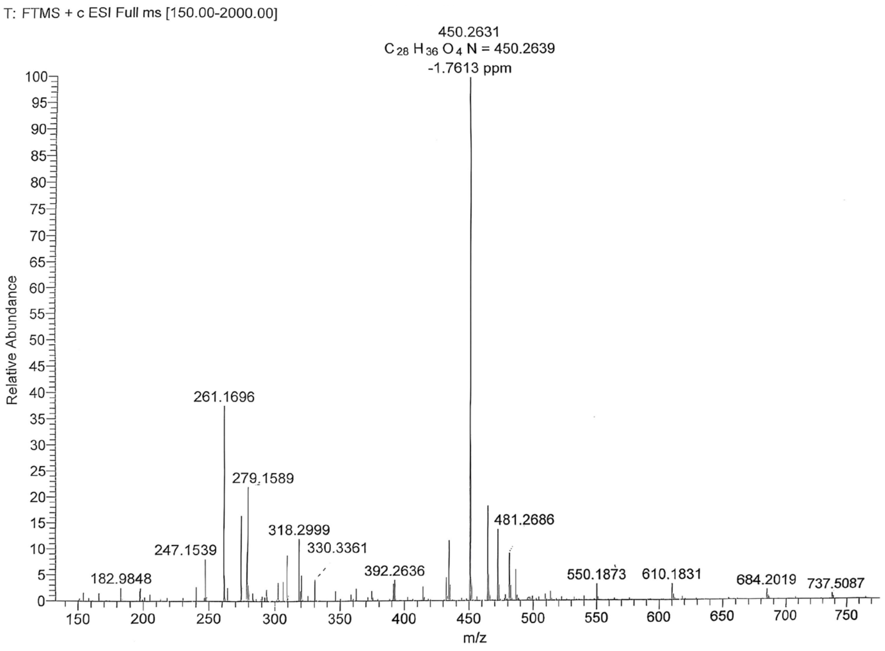

Figure S11. IR spectra of compound **2**.

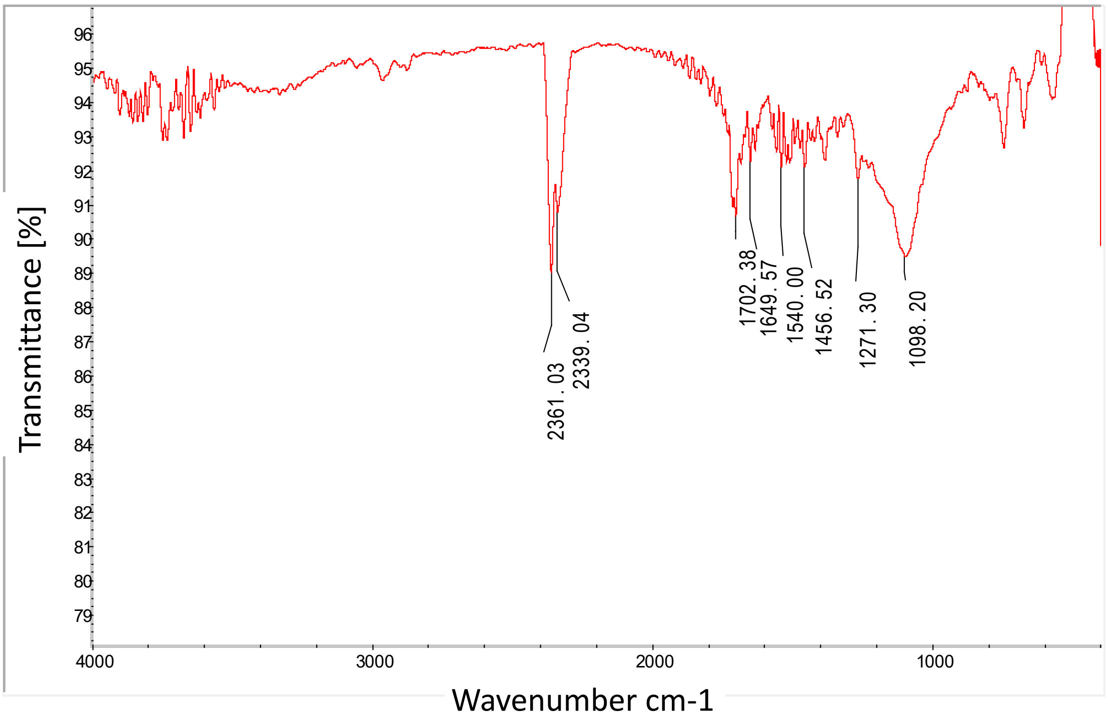

Figure S12. The ^{1}H NMR spectrum of compound **2** in $CDCl_3$.

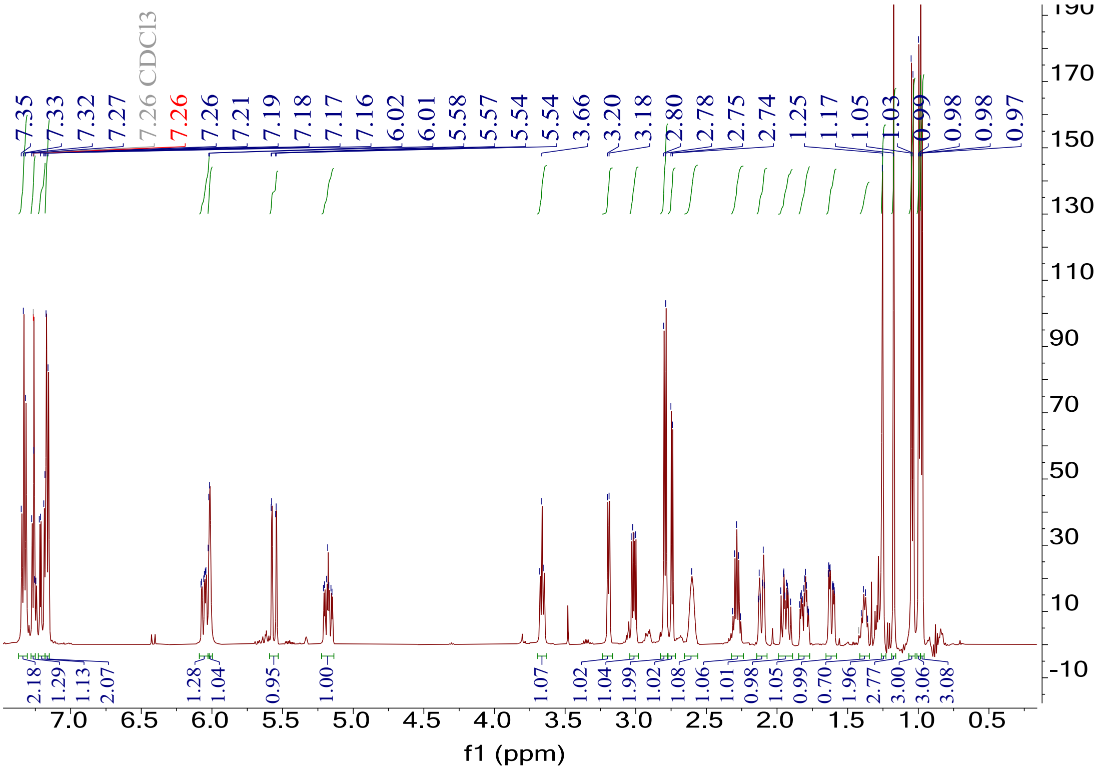

Figure S13. The ^{13}C NMR spectrum of compound **2** in $CDCl_3$.

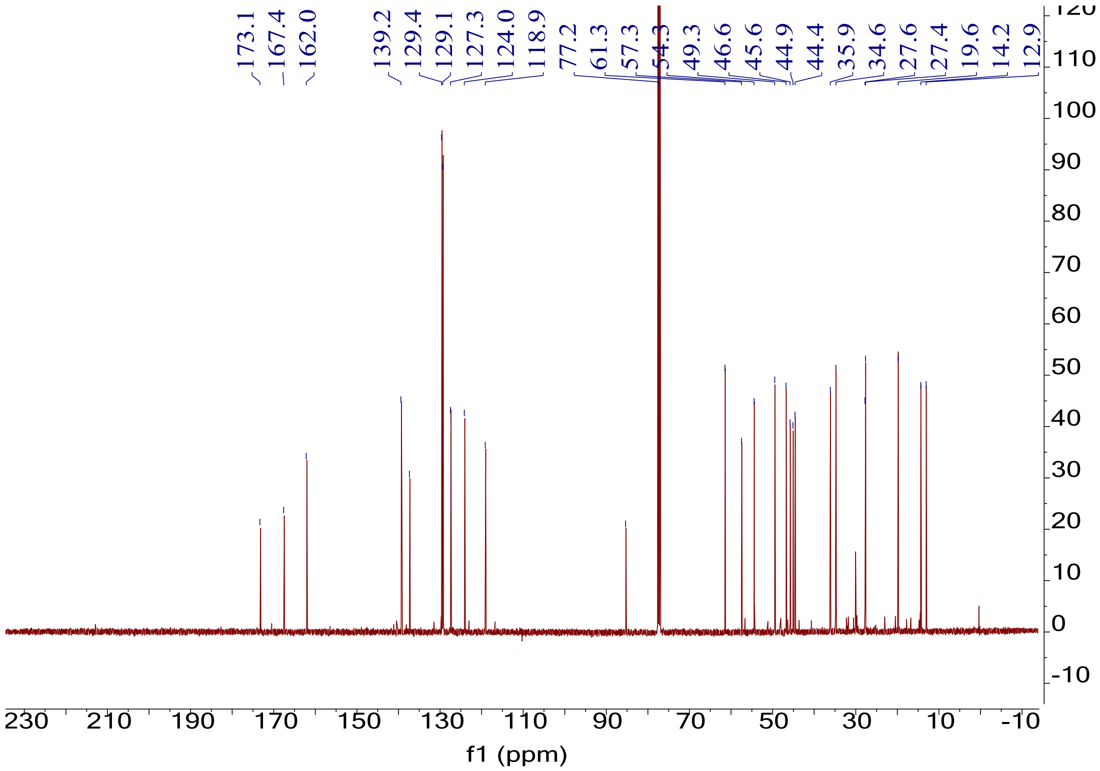

Figure S14. The HSQC spectrum of compound **2** in $CDCl_3$.

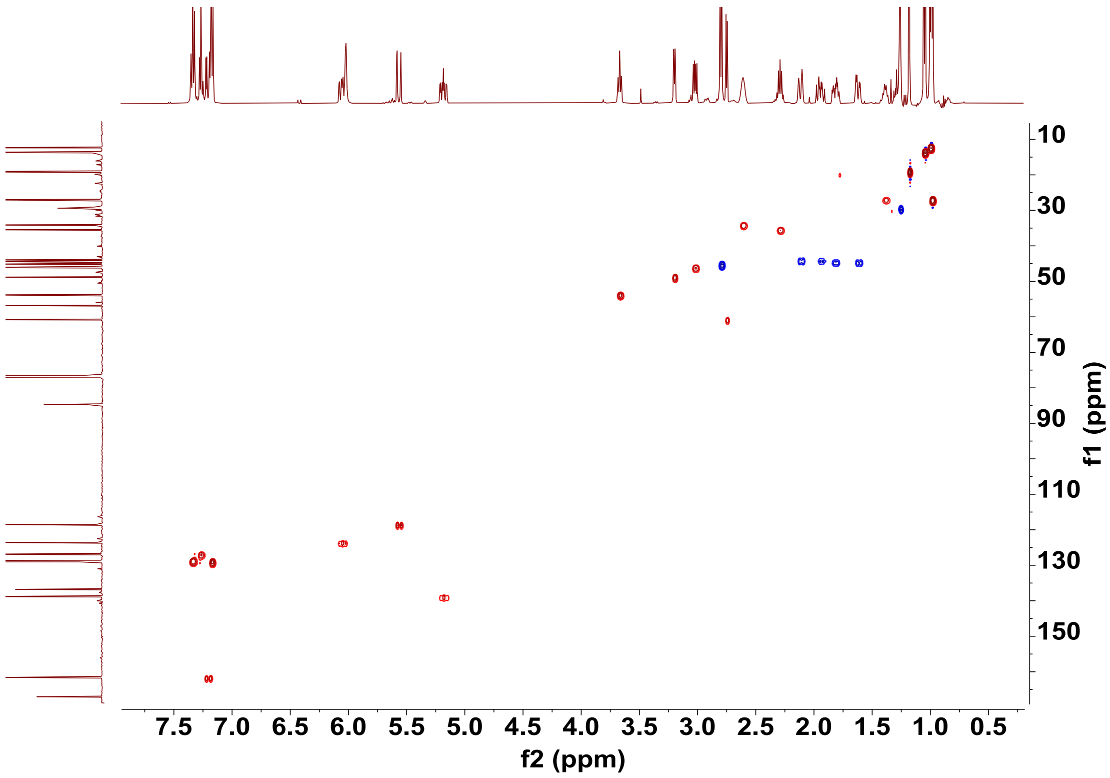

Figure S15. The HMBC spectrum of compound **2** in $CDCl_3$.

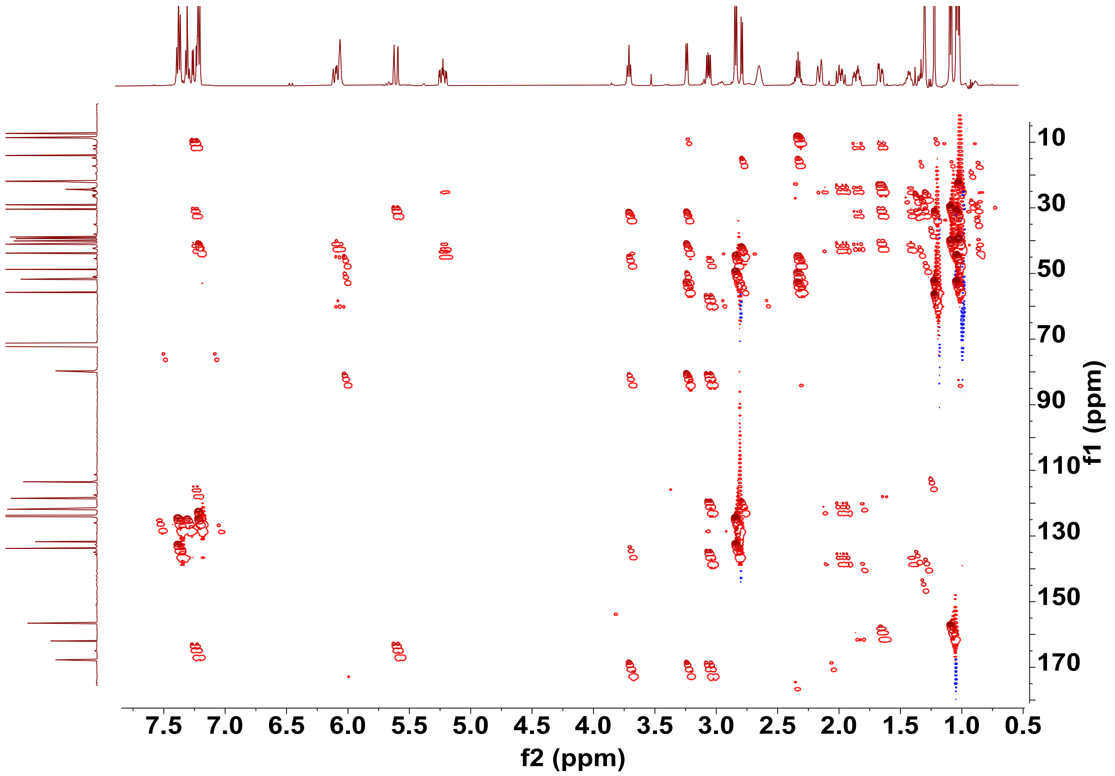

Figure S16. The ^{1}H–^{1}H COSY spectrum of compound **2** in $CDCl_3$.

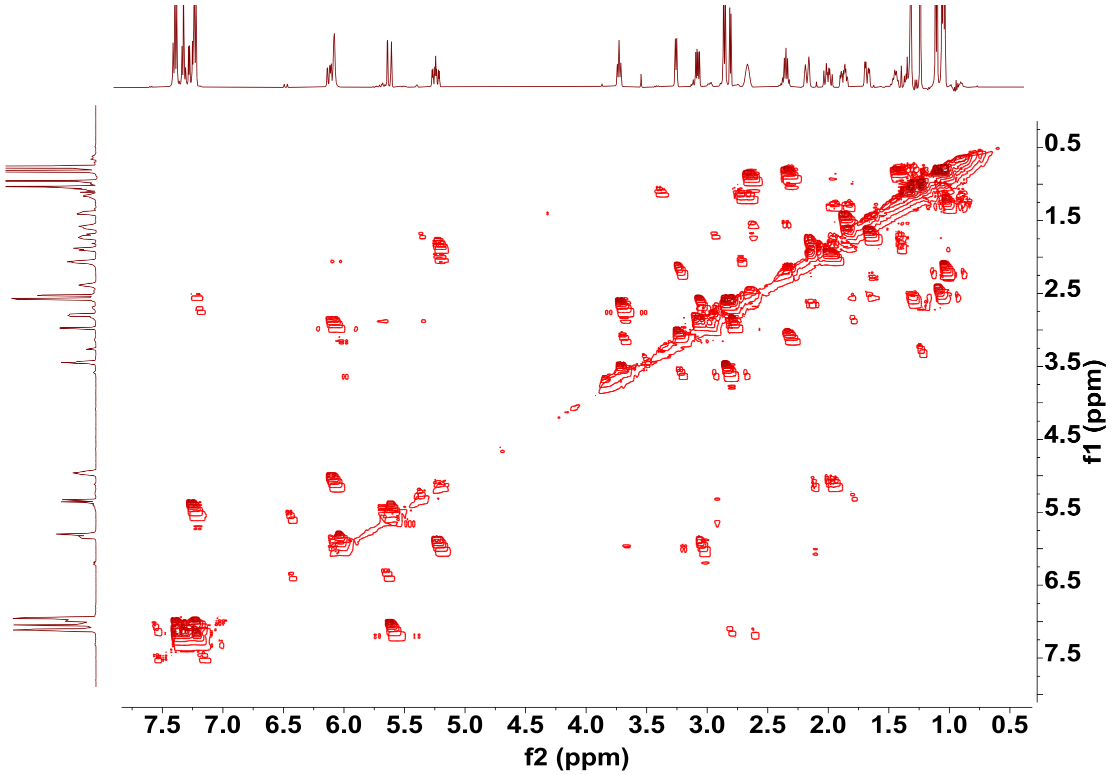

Figure S17. The NOESY spectrum of compound **2** in $CDCl_3$.

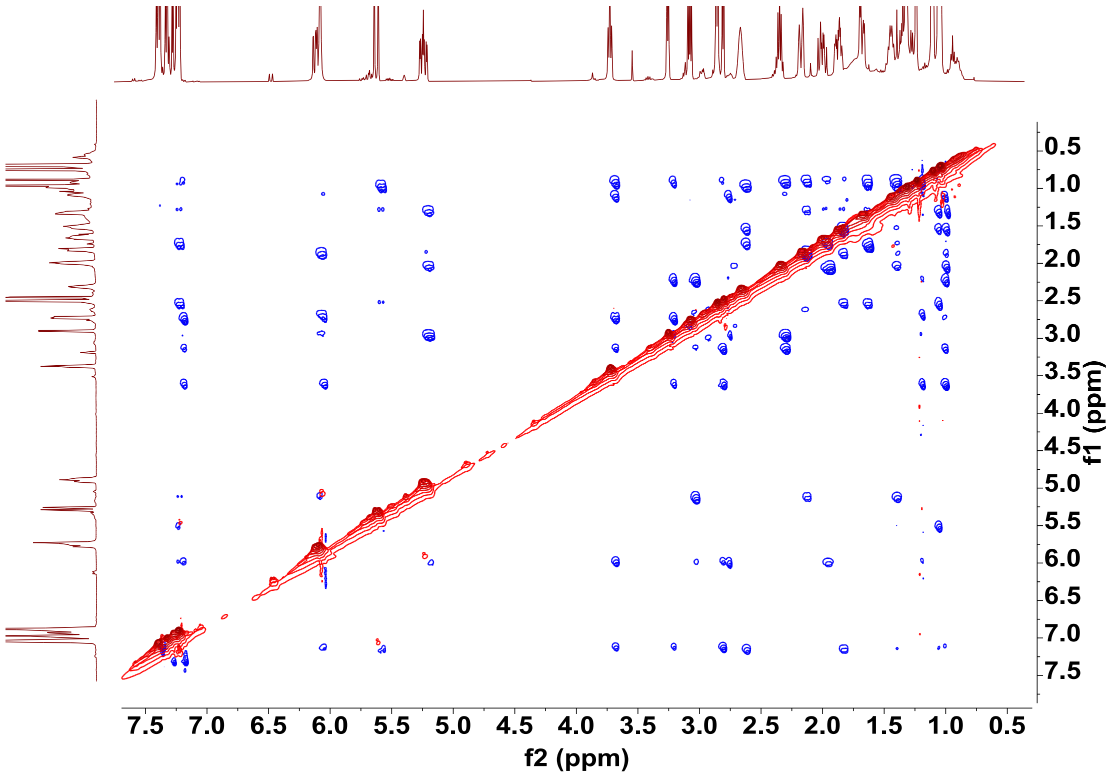

Figure S18. HR-ESI-MS spectra of compound **3**.

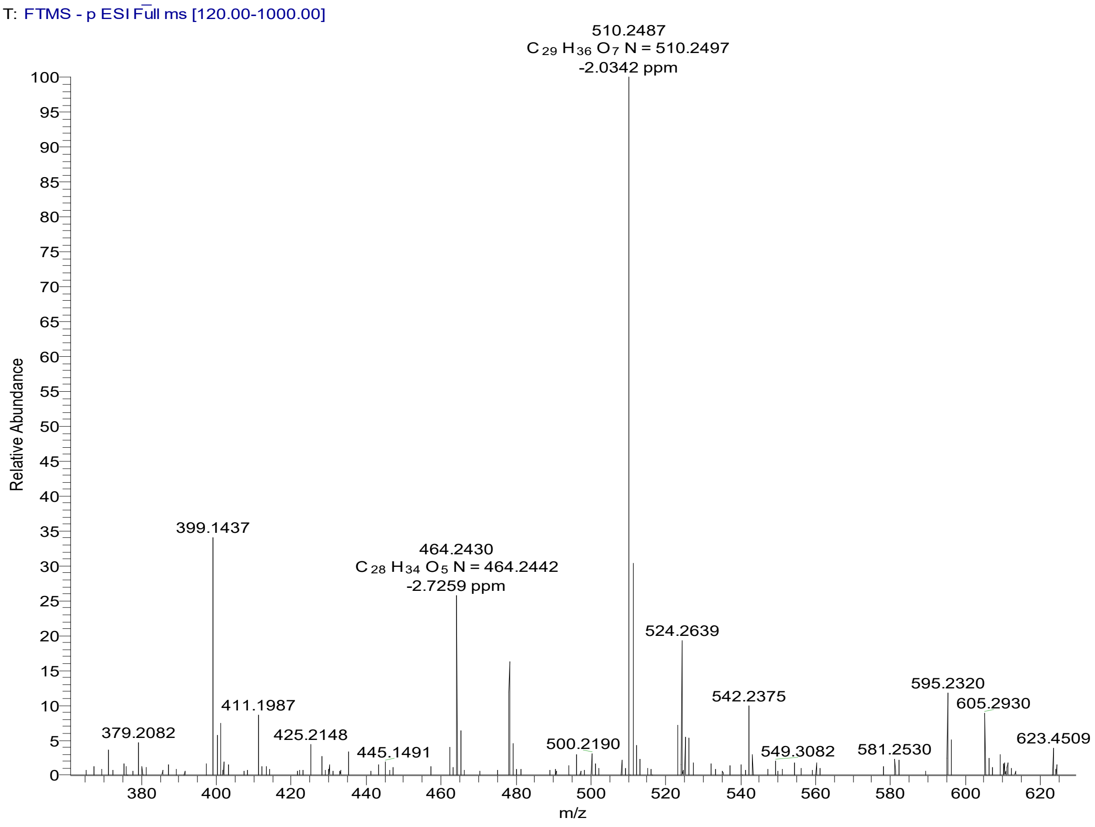

Figure S19. IR spectra of compound **3**.

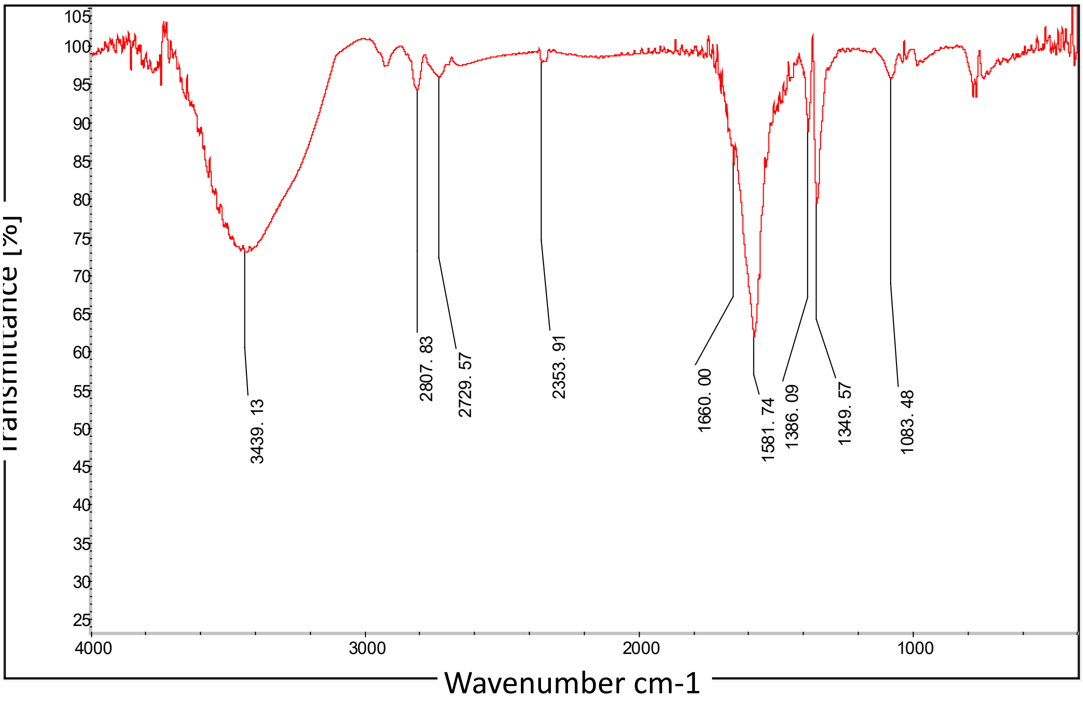

Figure S20. The ^{1}H NMR spectrum of compound **3** in $CDCl_3$.

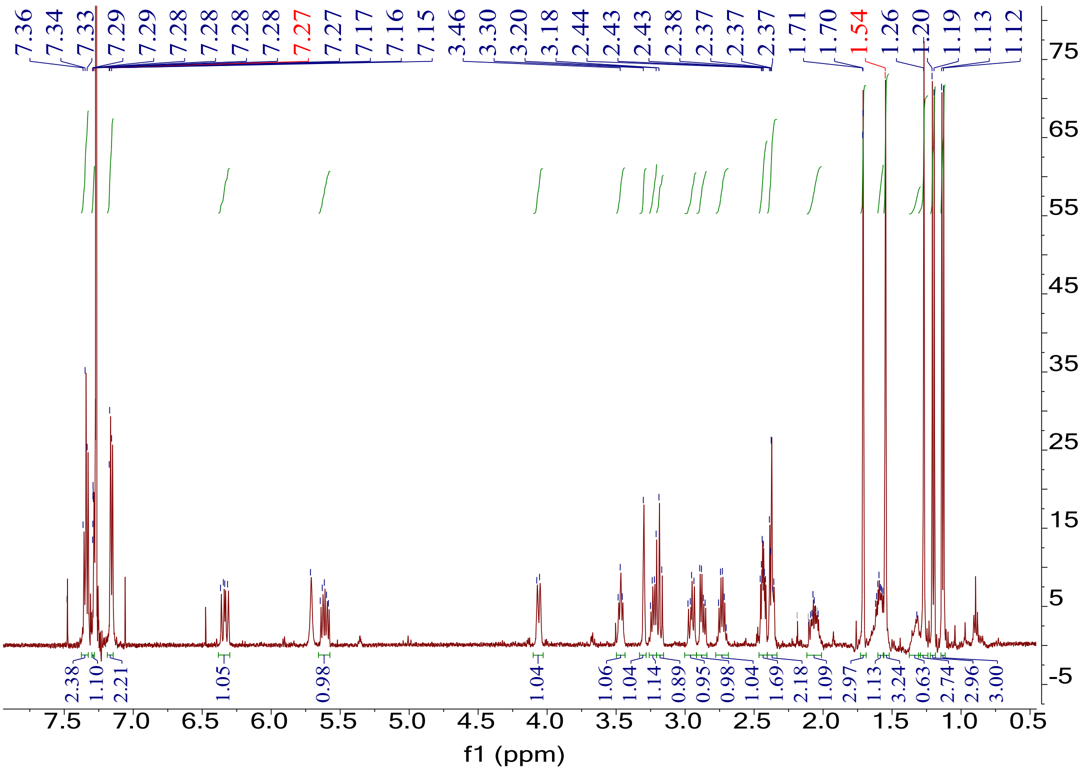

Figure S21. The ^{13}C NMR spectrum of compound **3** in $CDCl_3$.

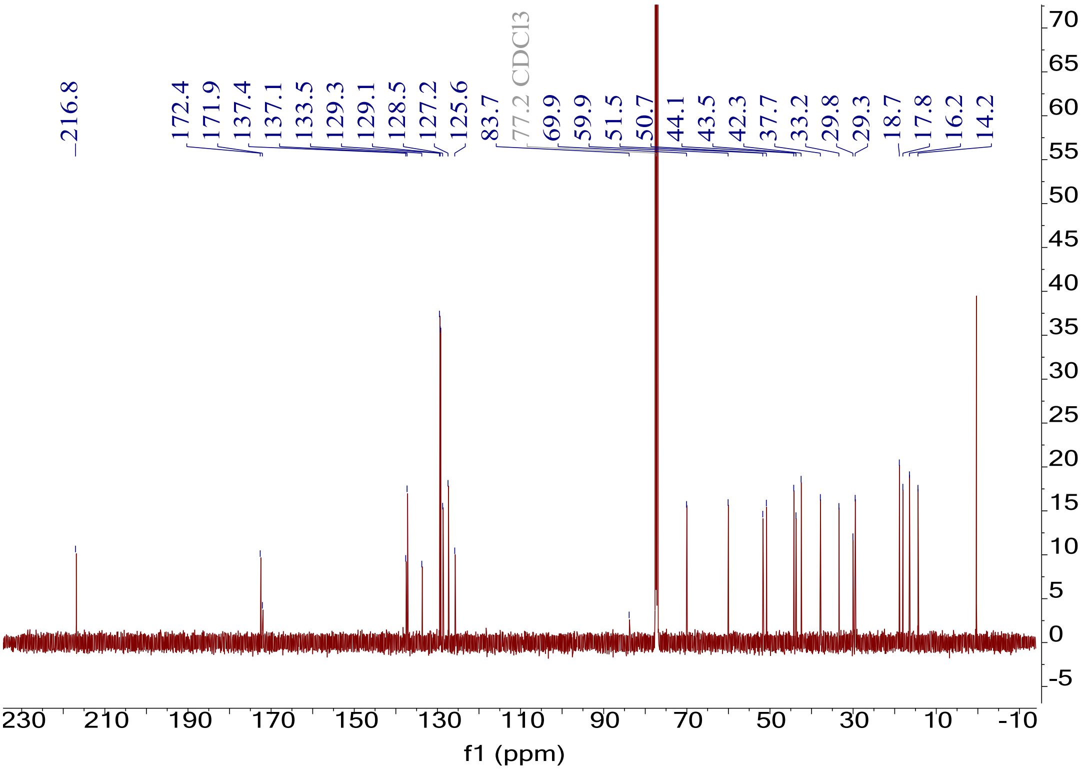

Figure S22. The HSQC spectrum of compound **3** in $CDCl_3$.

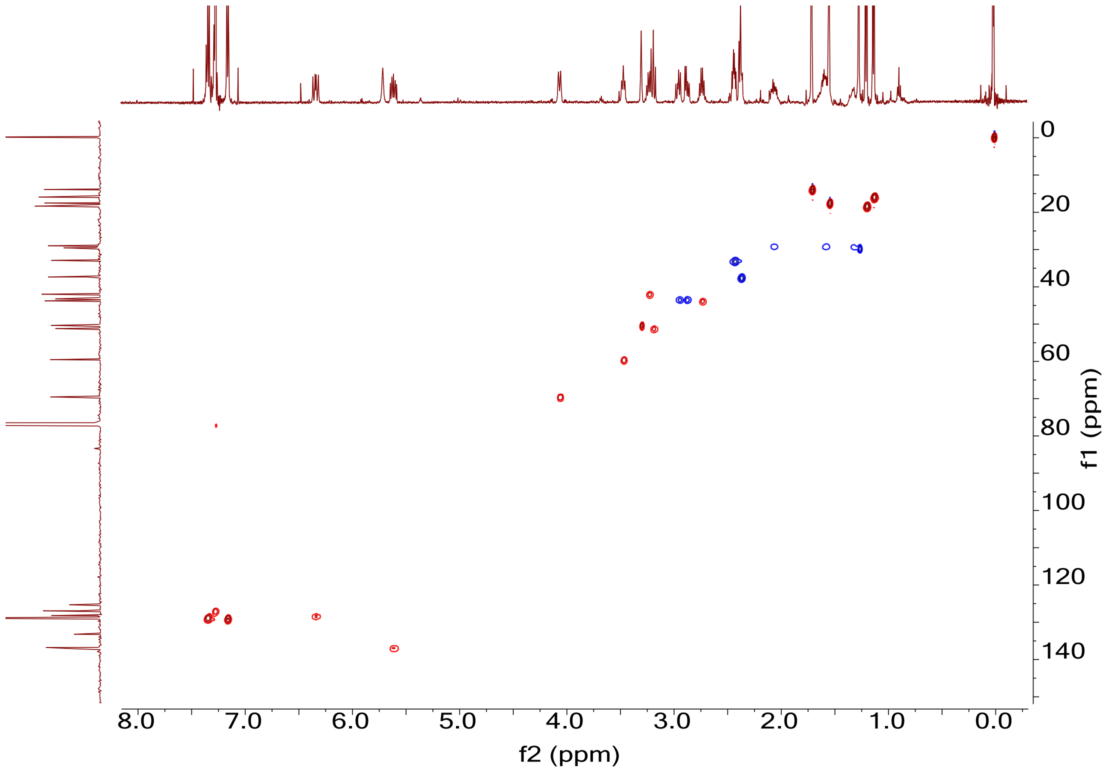

Figure S23. The HMBC spectrum of compound **3** in $CDCl_3$.

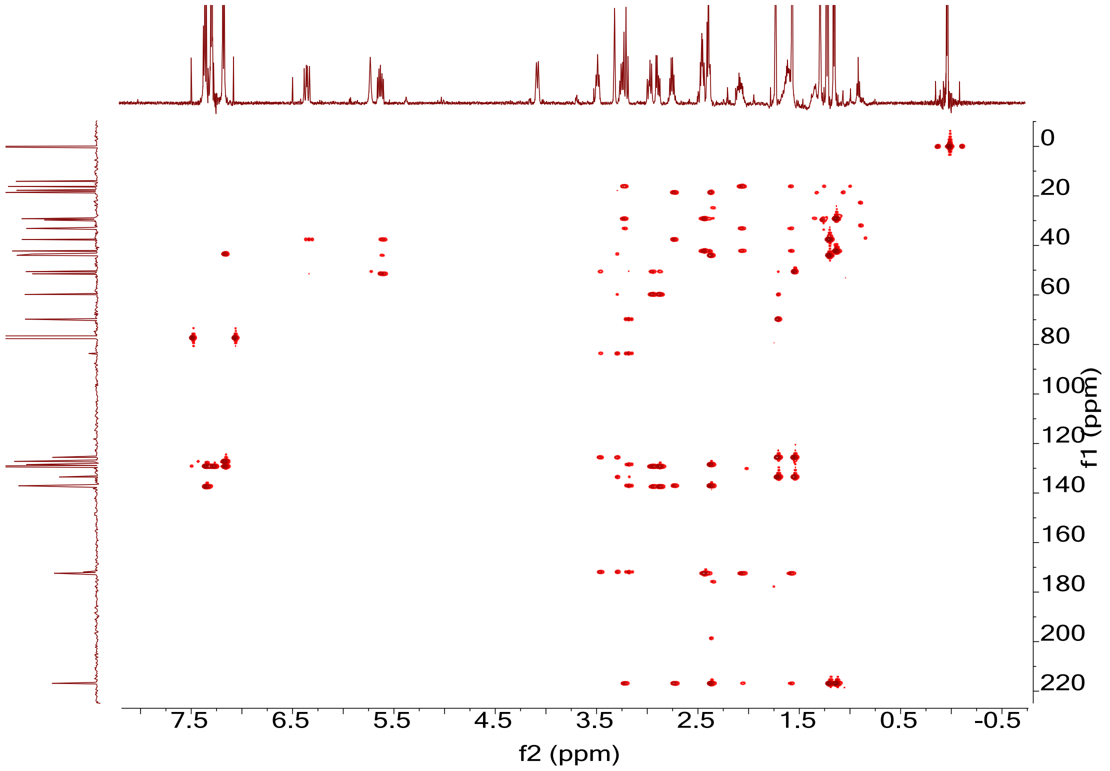

Figure S24. The ^{1}H–^{1}H COSY spectrum of compound **3** in $CDCl_3$.

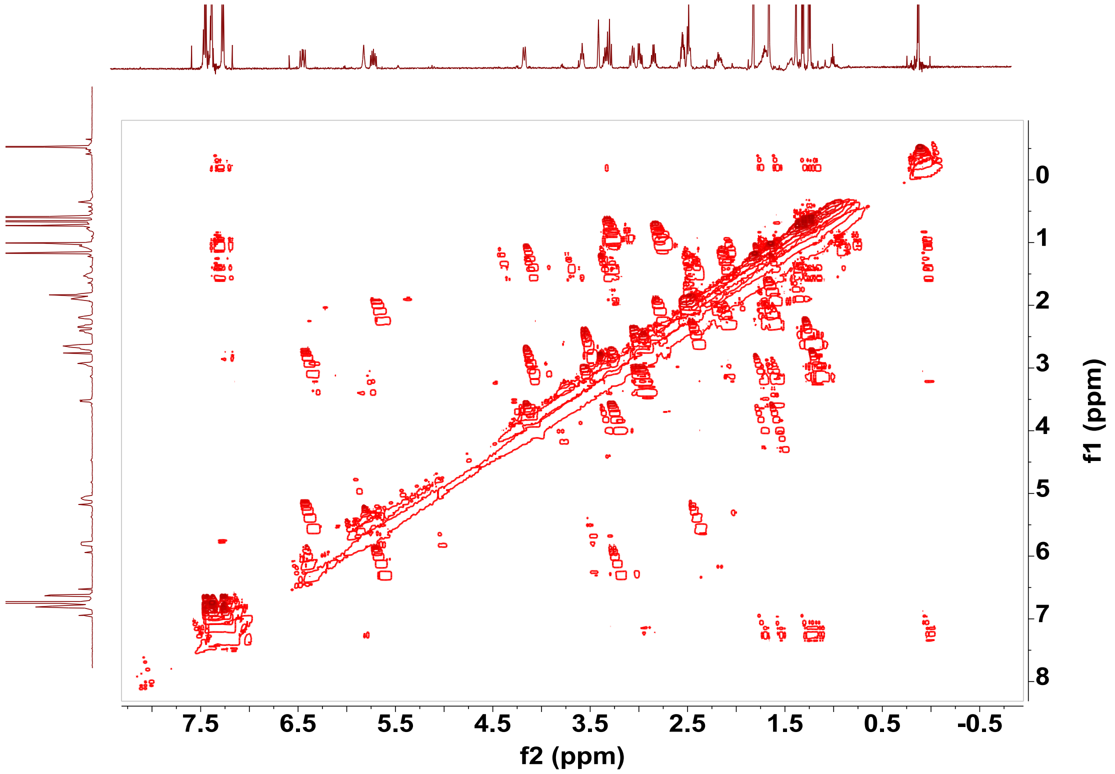

Figure S25. The NOESY spectrum of compound **3** in $CDCl_3$.

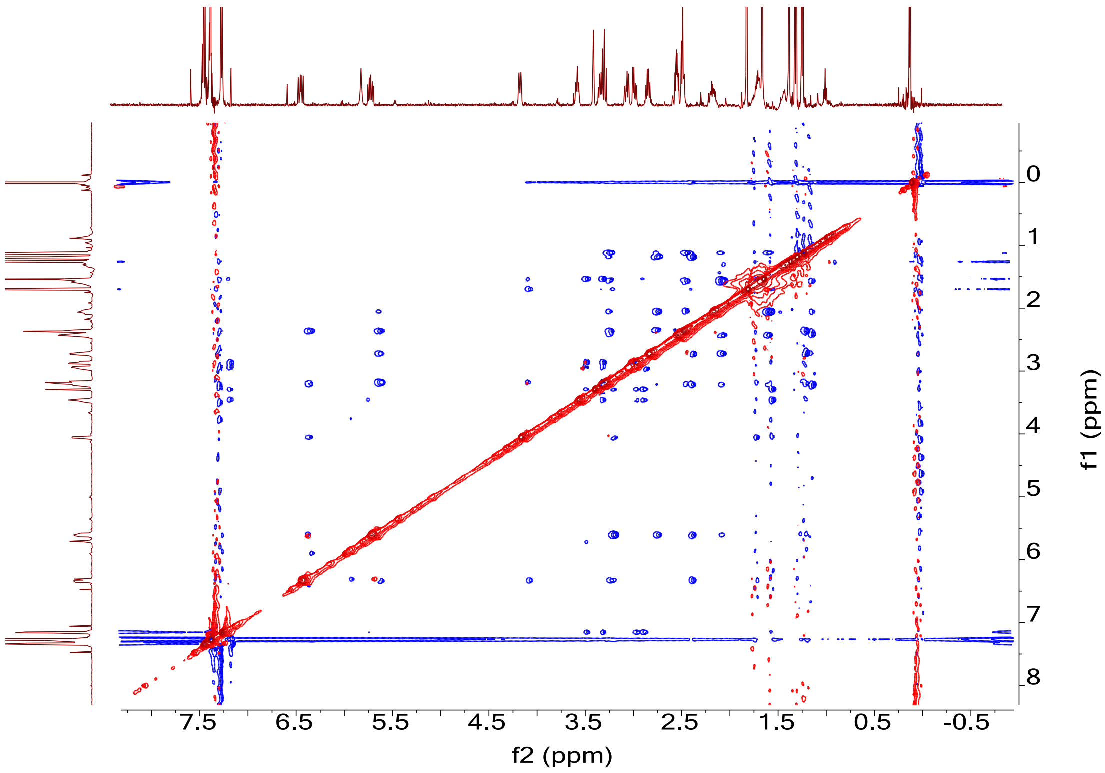

Figure S26. HR-ESI-MS spectra of compound **4**.

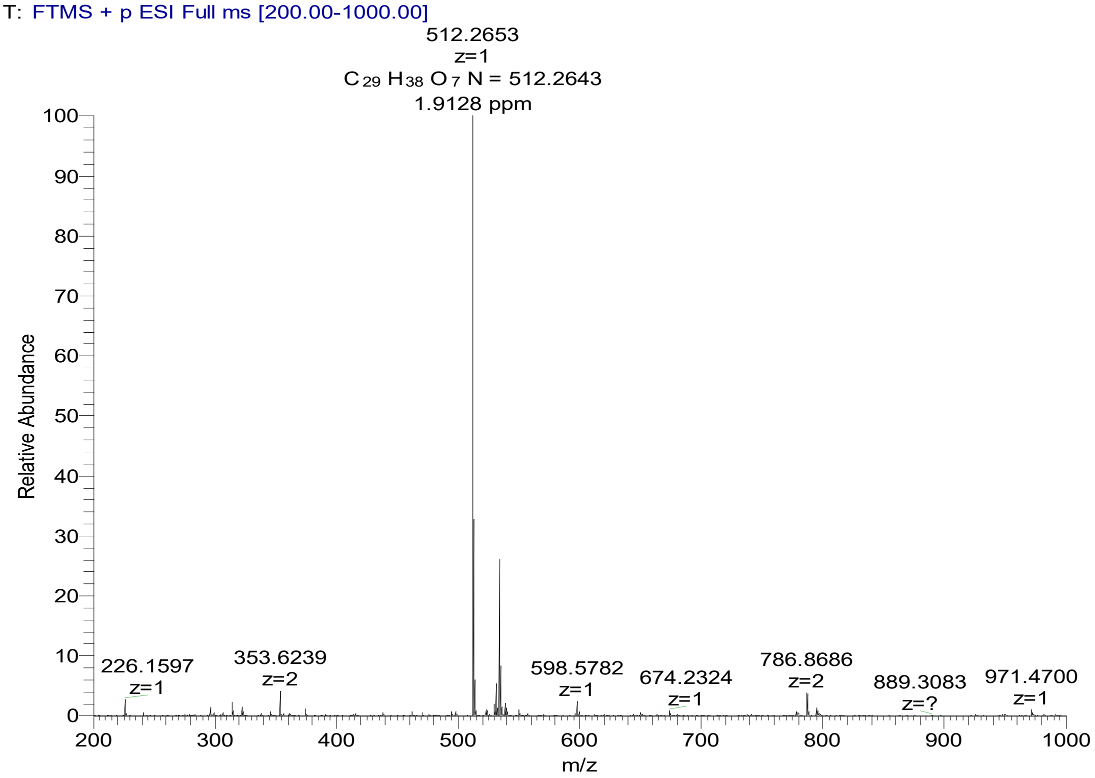

Figure S27. IR spectra of compound **4**.

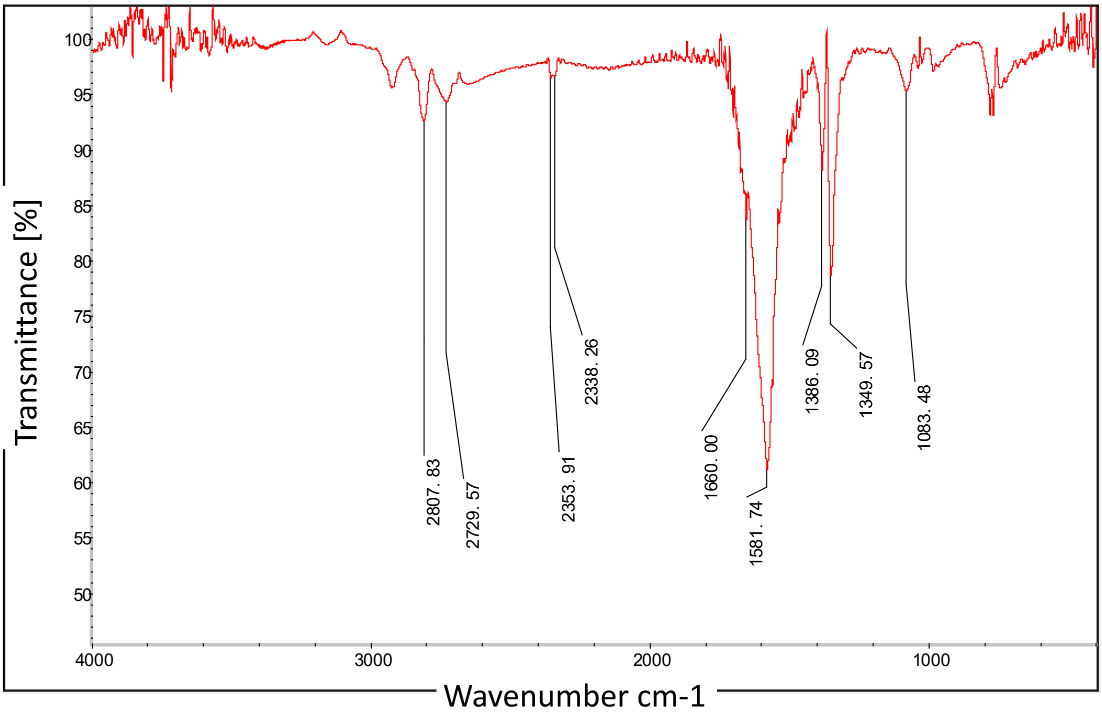

Figure S28. The ^{1}H NMR spectrum of compound **4** in $CDCl_3$.

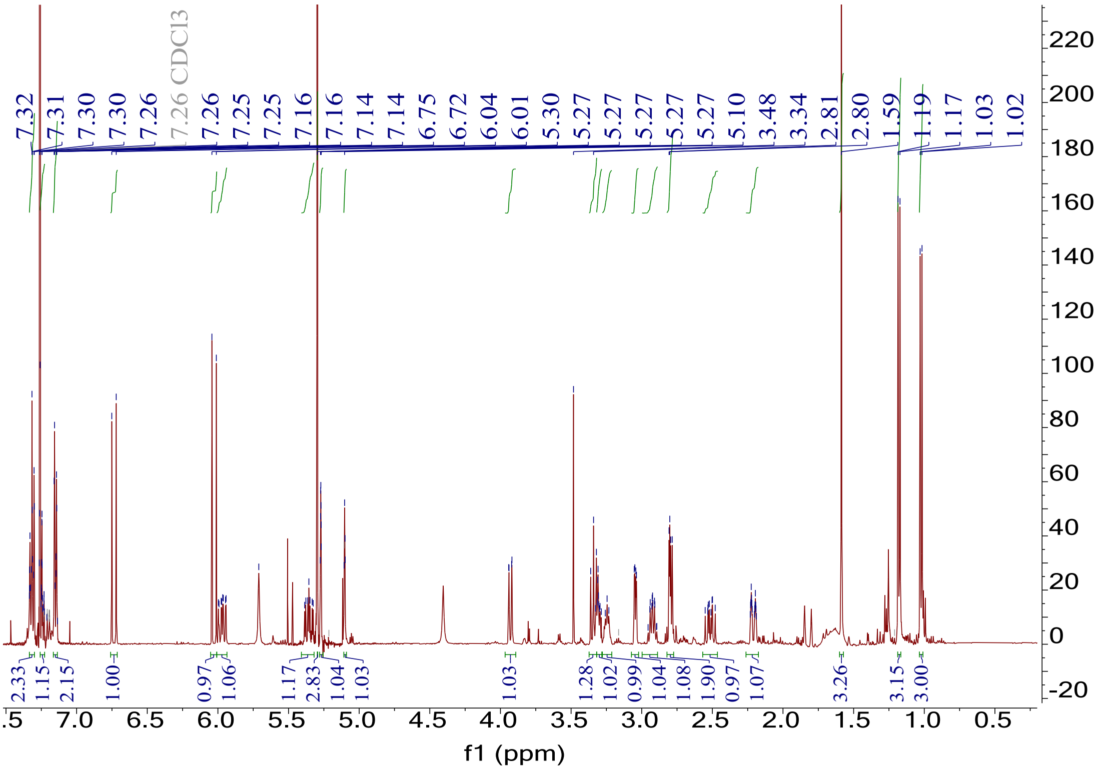

Figure S29. The ^{13}C NMR spectrum of compound **4** in $CDCl_3$.

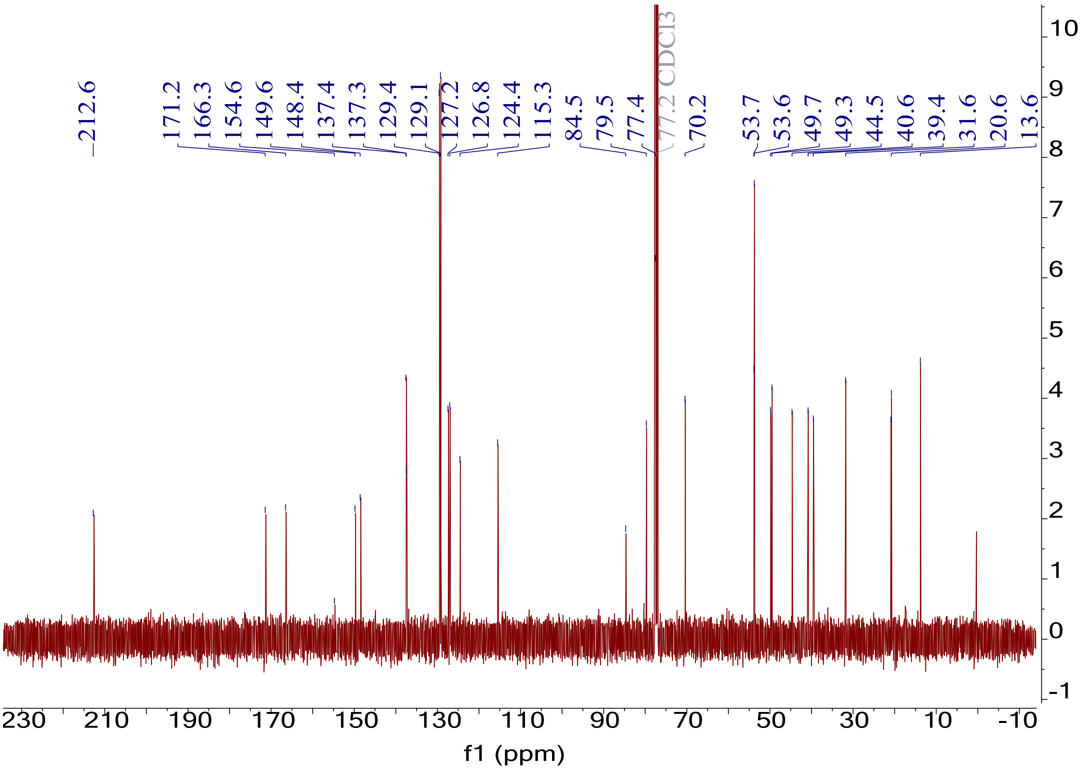

Figure S30. The HSQC spectrum of compound **4** in $CDCl_3$.

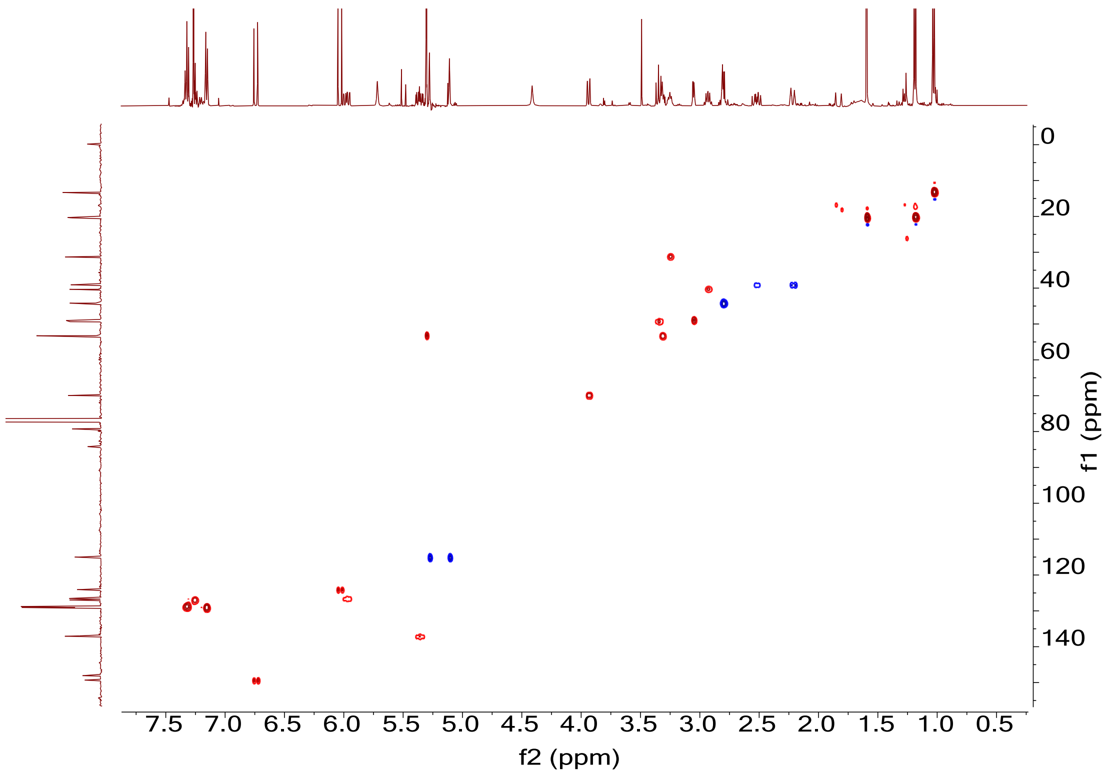

Figure S31. The HMBC spectrum of compound **4** in $CDCl_3$.

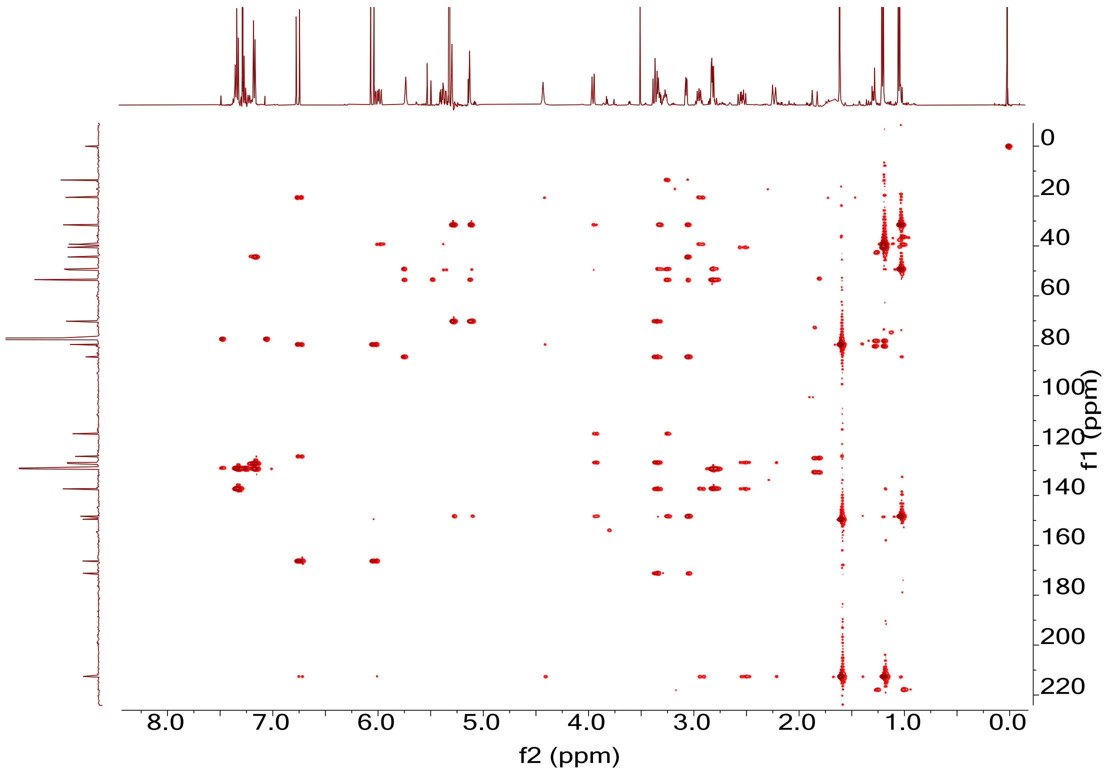

Figure S32. The ^1H–^1H COSY spectrum of compound **4** in $CDCl_3$.

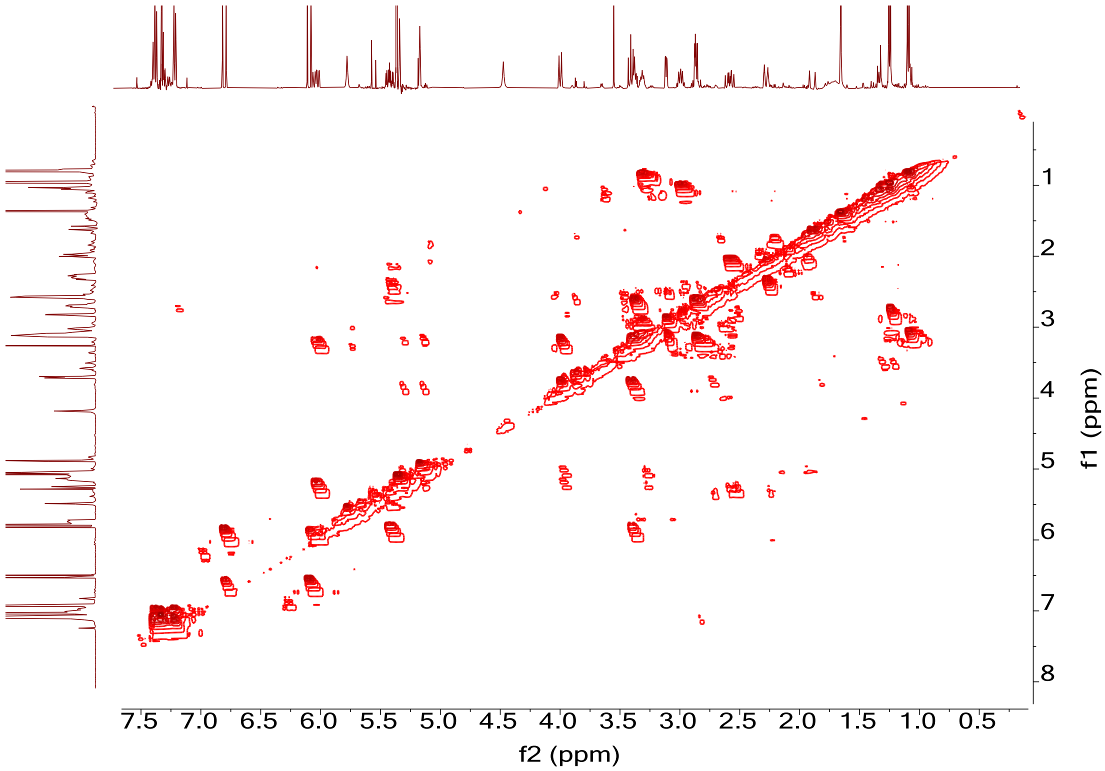

Figure S33. The NOESY spectrum of compound **4** in $CDCl_3$.

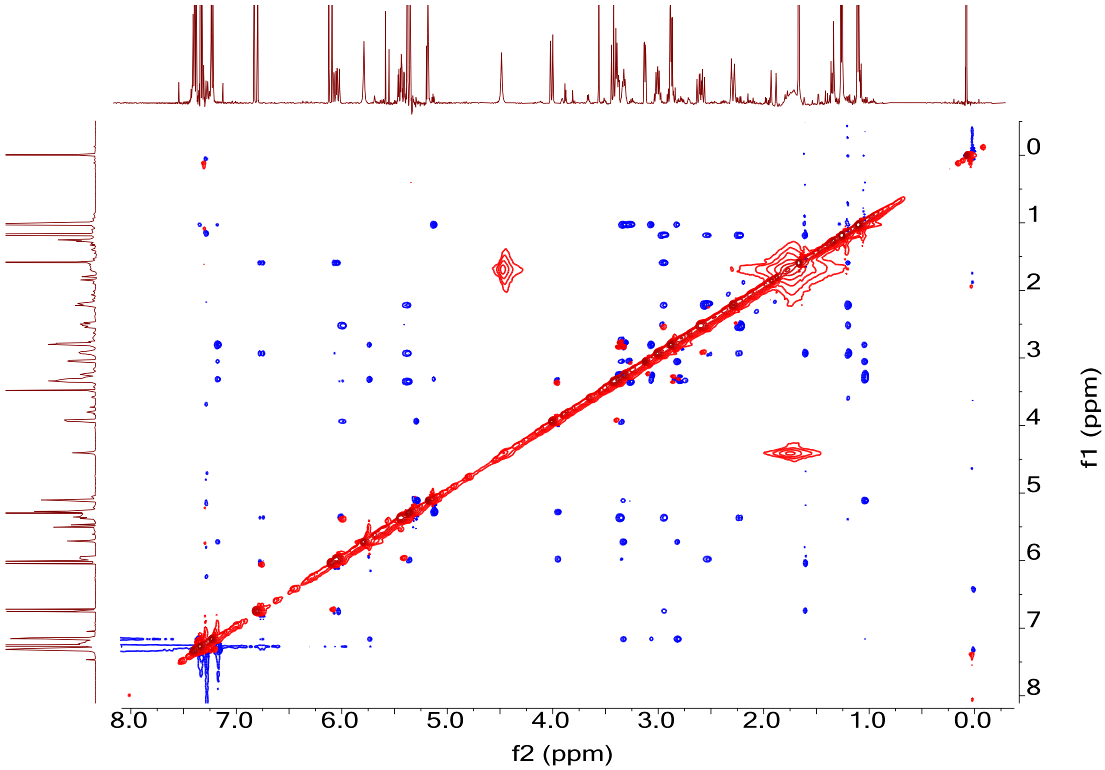

Figure S34. HR-ESI-MS spectra of compound **5**.

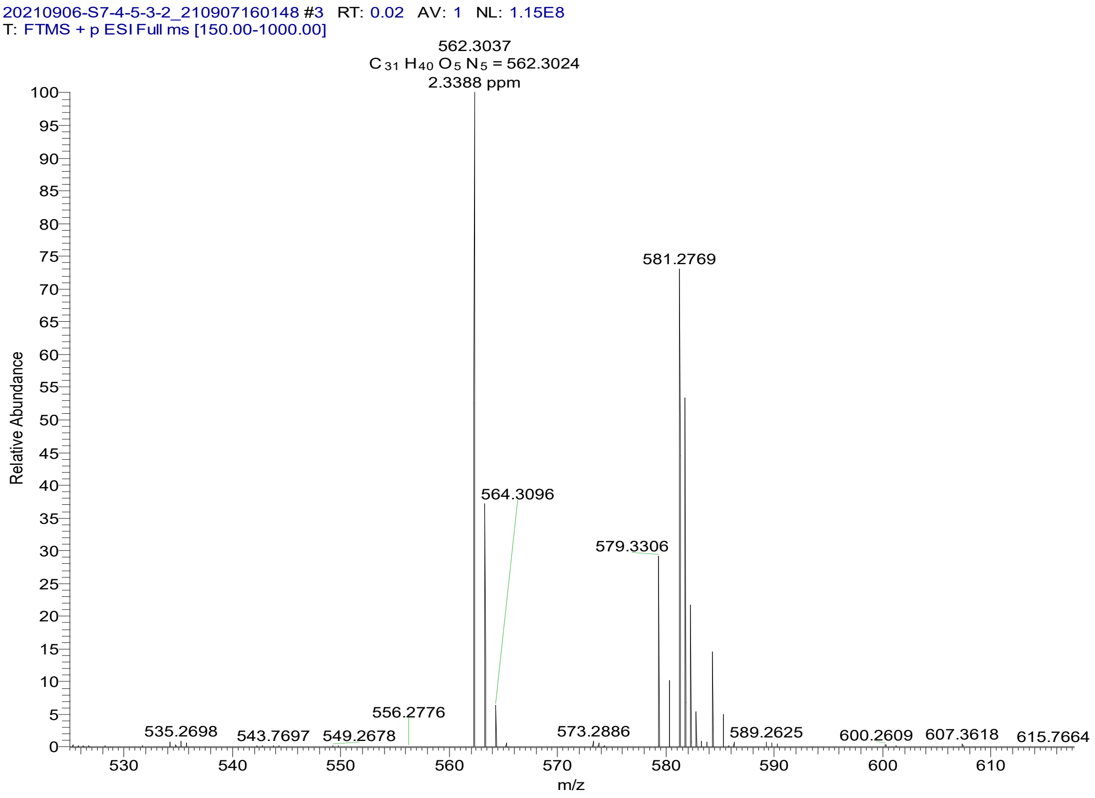

Figure S35. IR spectra of compound **5**.

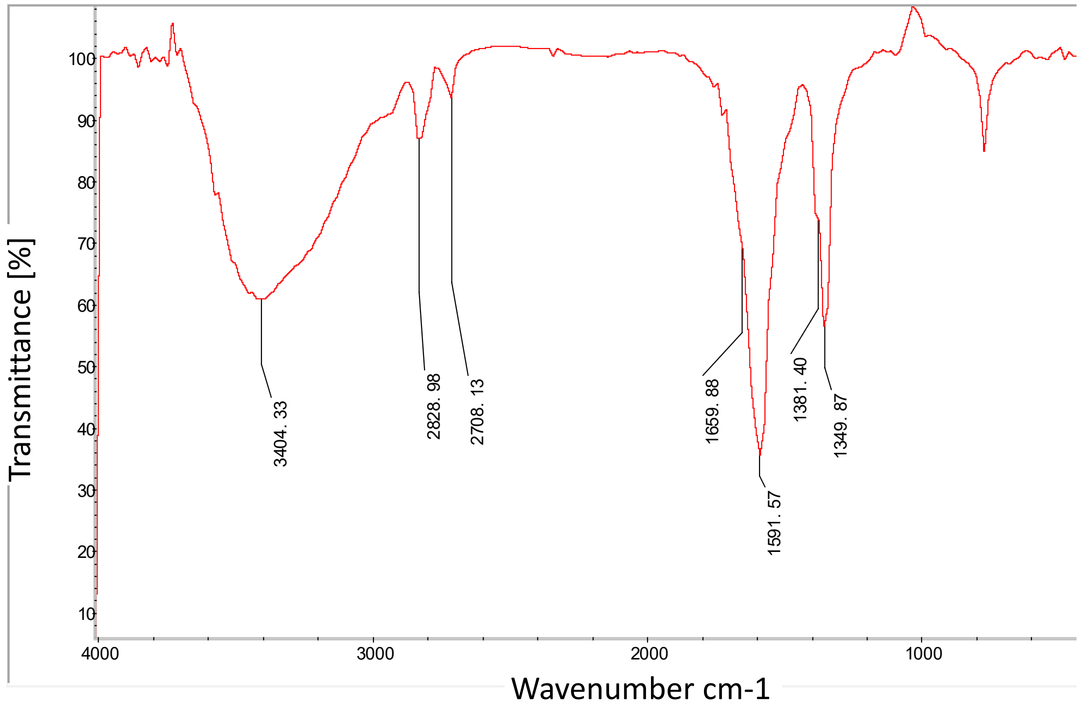

Figure S36. The ^{1}H NMR spectrum of compound **5** in C_6D_6.

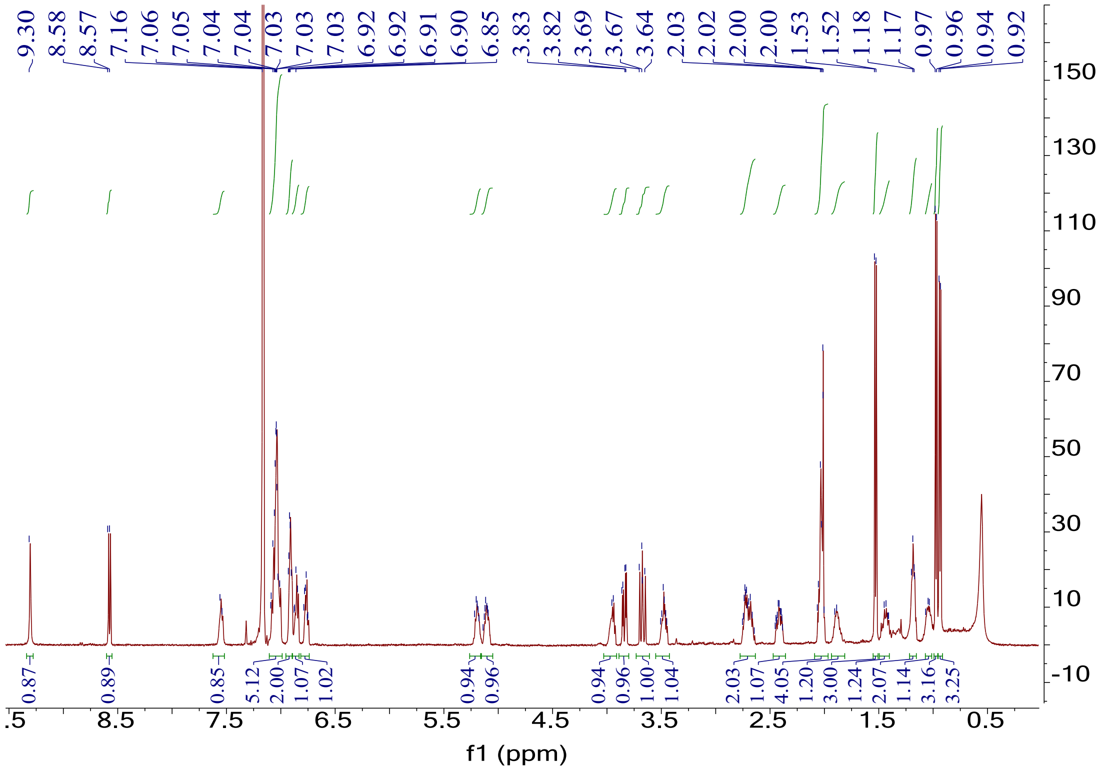

Figure S37. The ^{13}C NMR spectrum of compound **5** in C_6D_6.

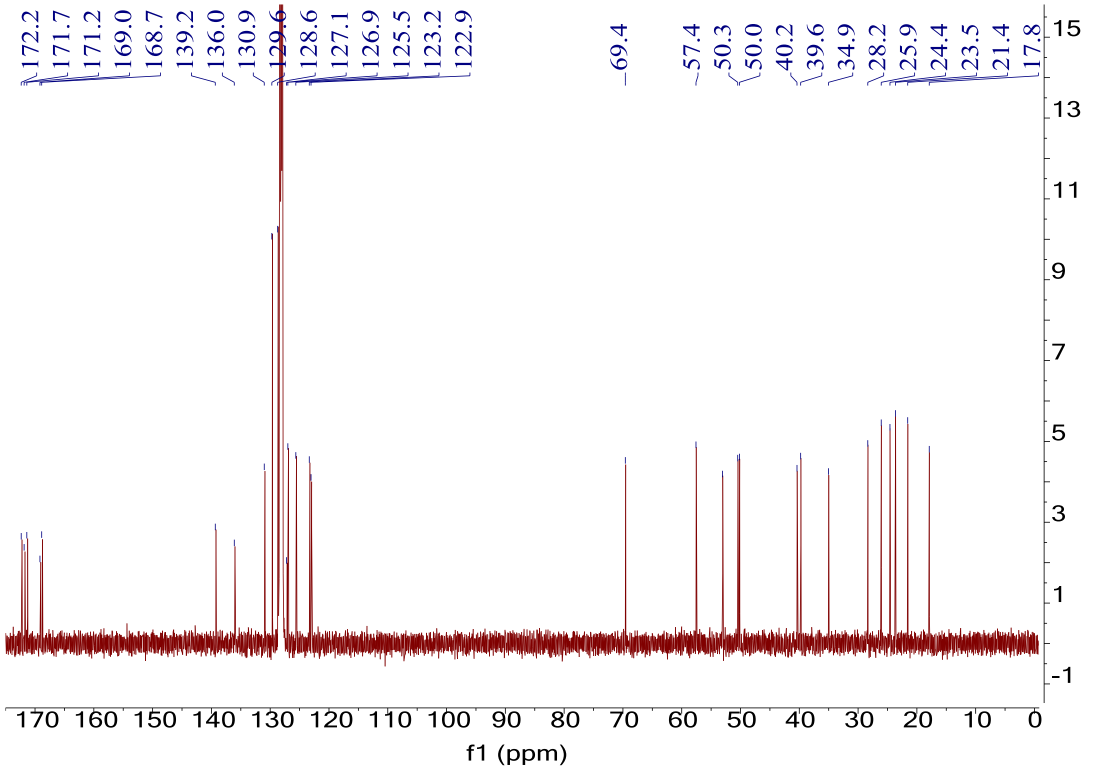

Figure S38. The HSQC spectrum of compound **5** in C_6D_6.

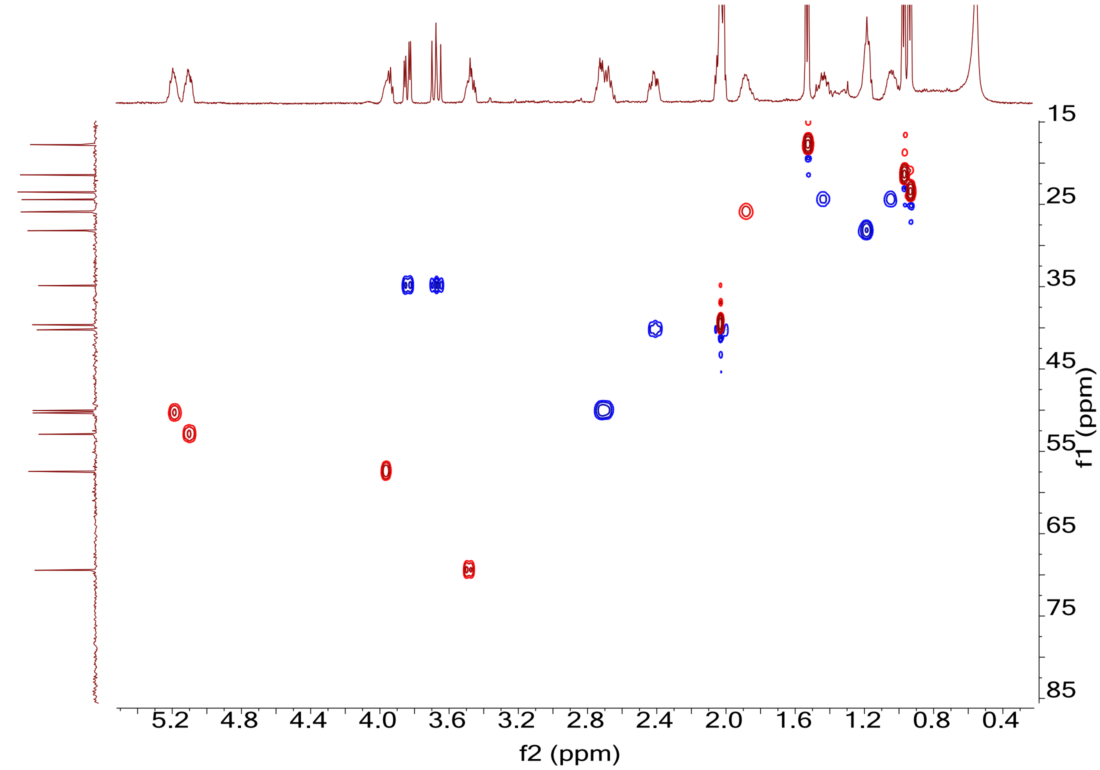

Figure S39. The HMBC spectrum of compound **5** in C_6D_6.

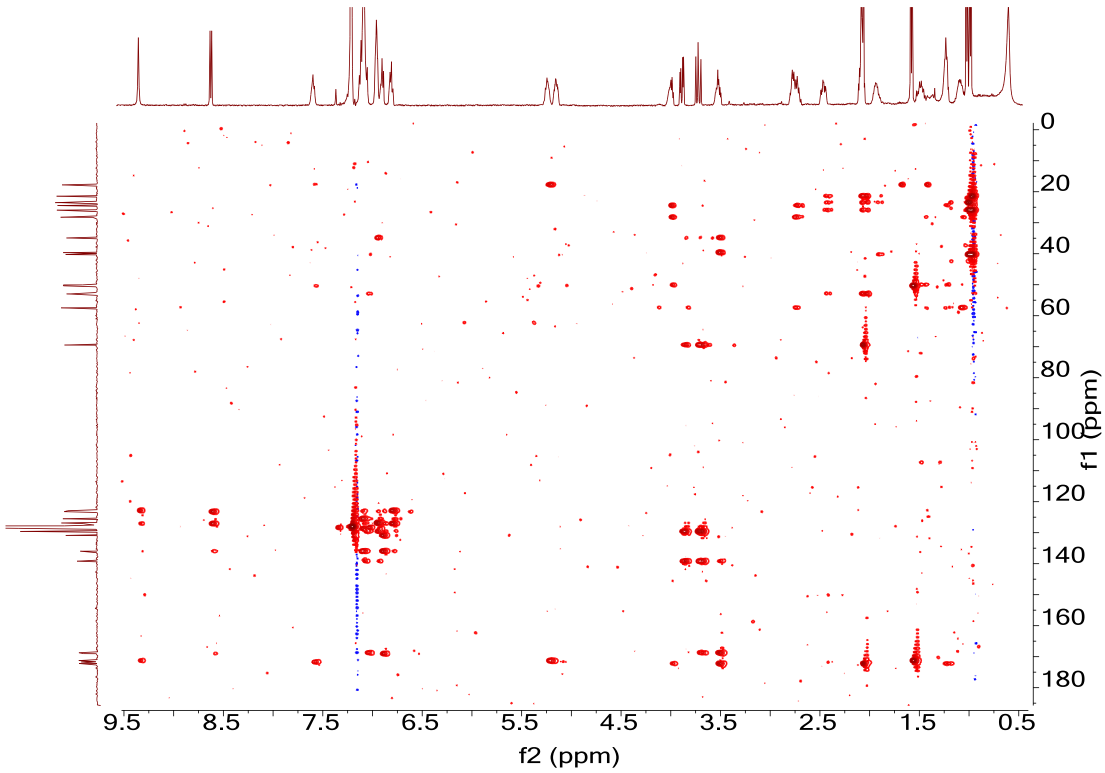

Figure S40. The ^{1}H–^{1}H COSY spectrum of compound **5** in C_6D_6.

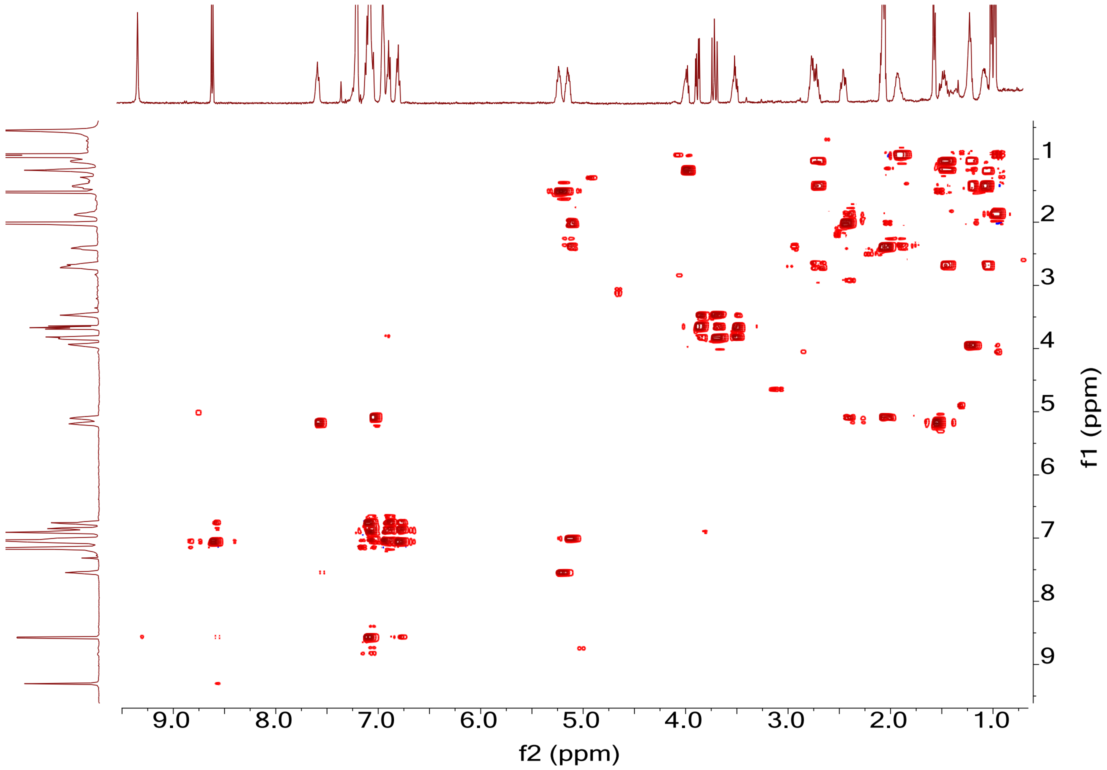

Figure S41. The **^{1}H** NMR spectrum of compound **6** in $CDCl_3$.

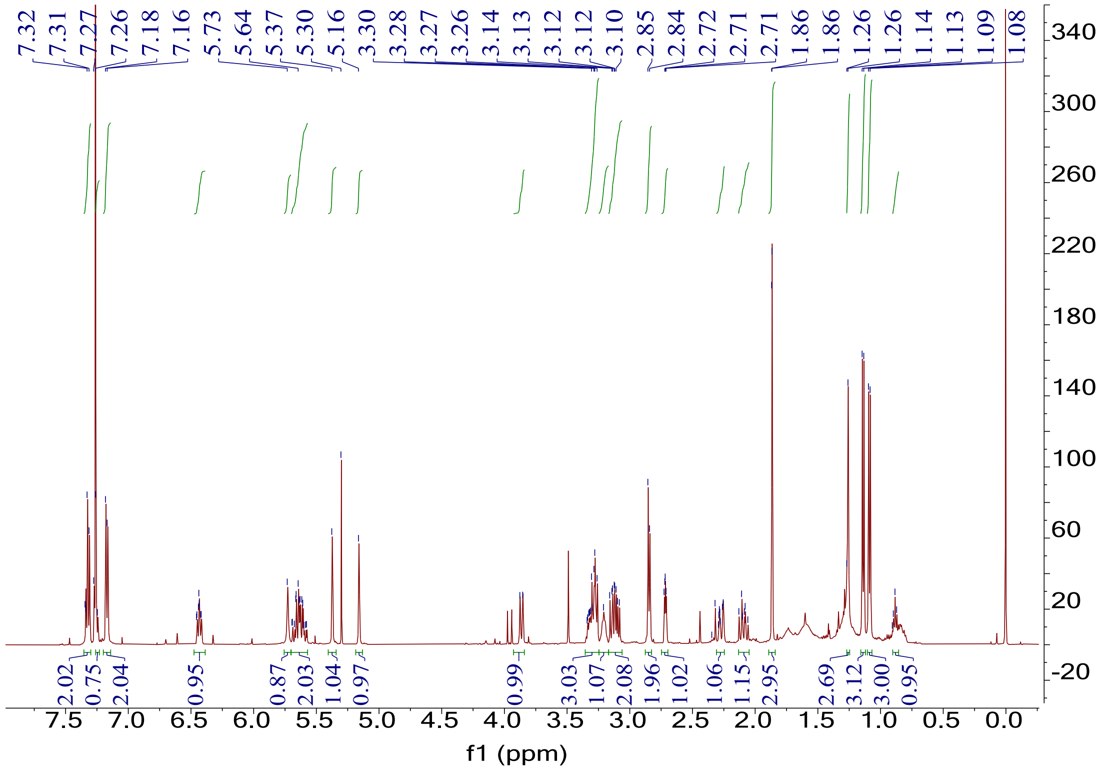

Figure S42. The ^{13}C NMR spectrum of compound **6** in $CDCl_3$.

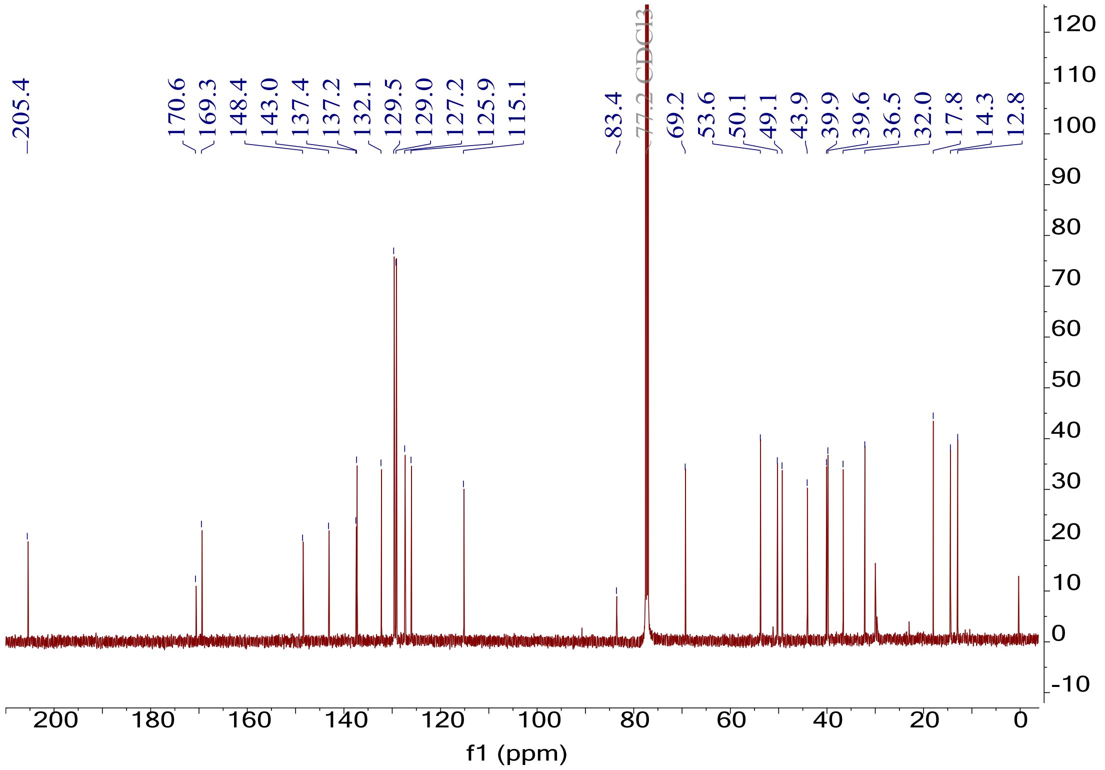

Figure S43. The ^{1}H NMR spectrum of compound **7** in $CDCl_3$.

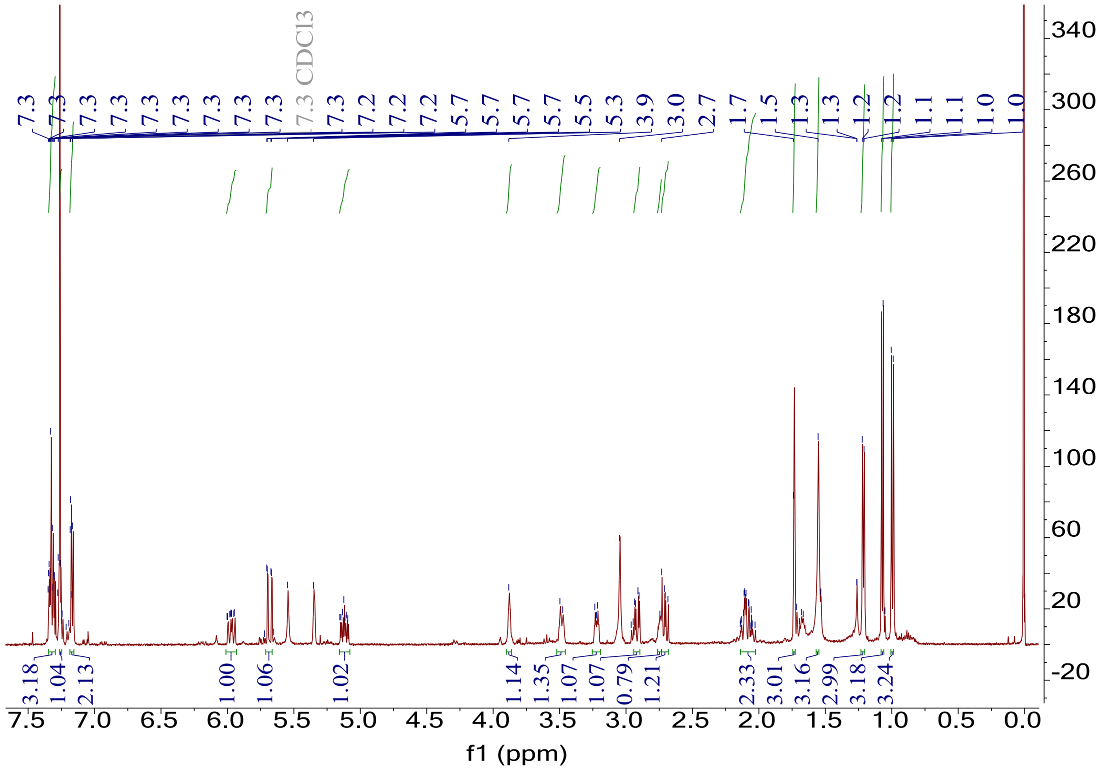

Figure S44. The ^{1}H NMR spectrum of compound **8** in $CDCl_3$.

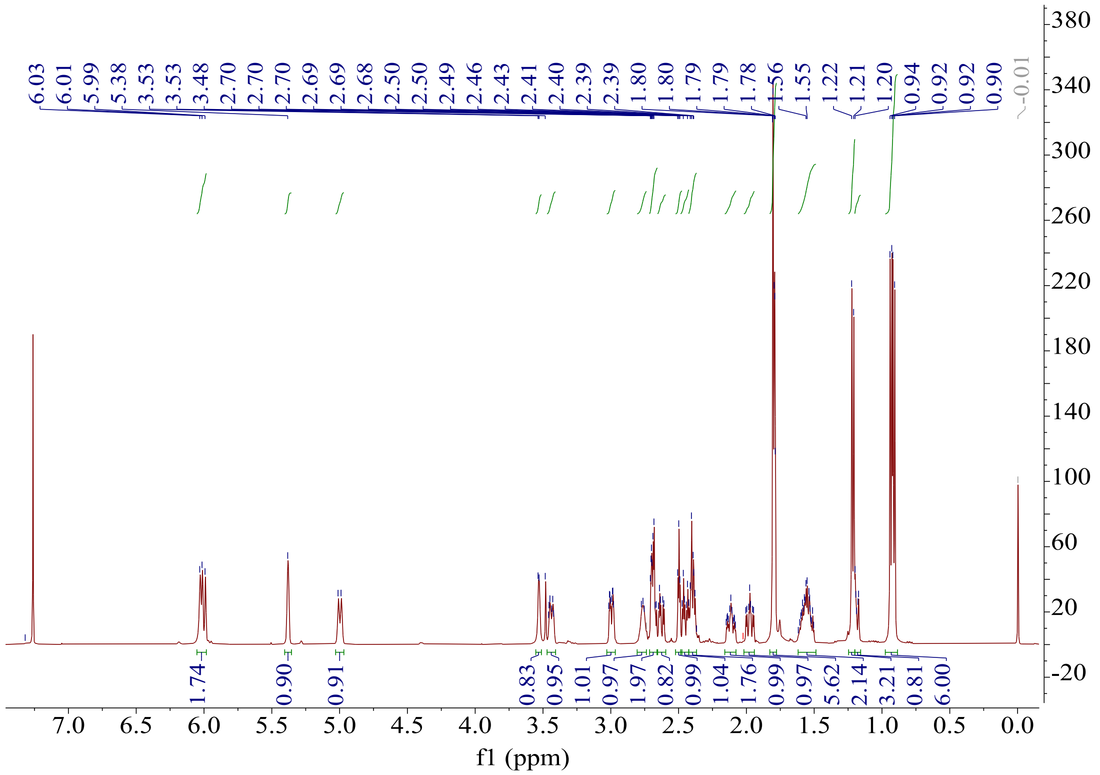

Figure S45. The ^{13}C NMR spectrum of compound **8** in $CDCl_3$.

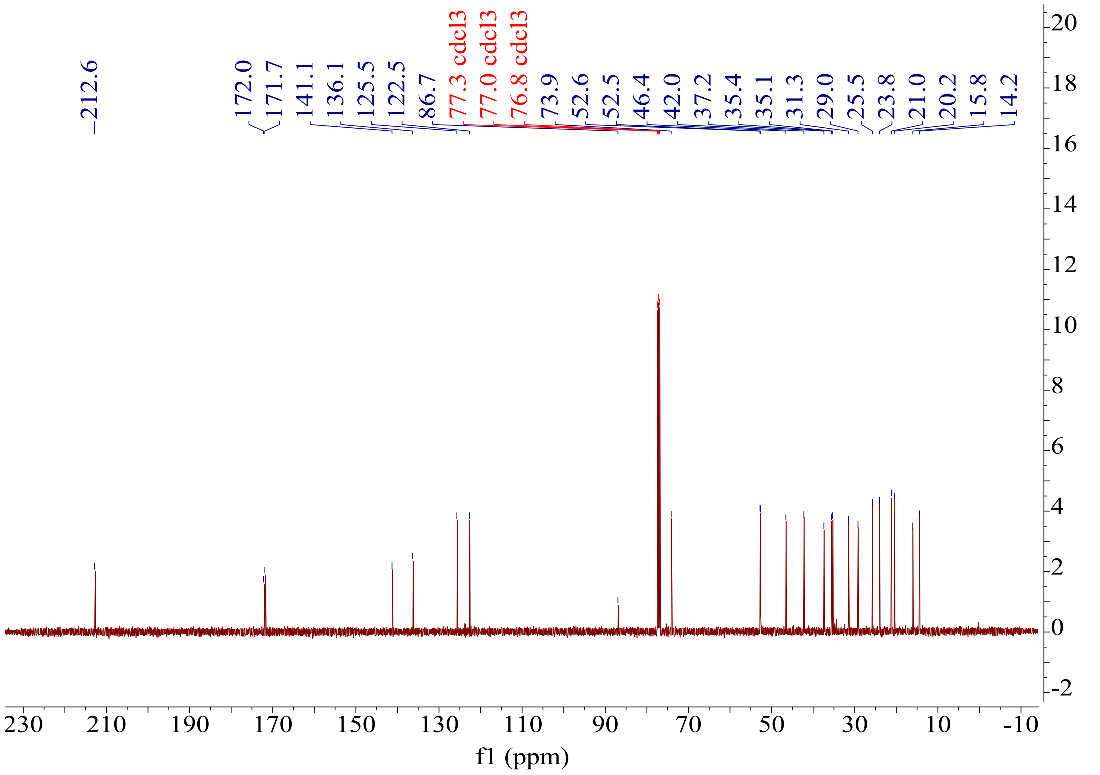

Figure S46. The ^{1}H NMR spectrum of compound **9** in $CDCl_3$.

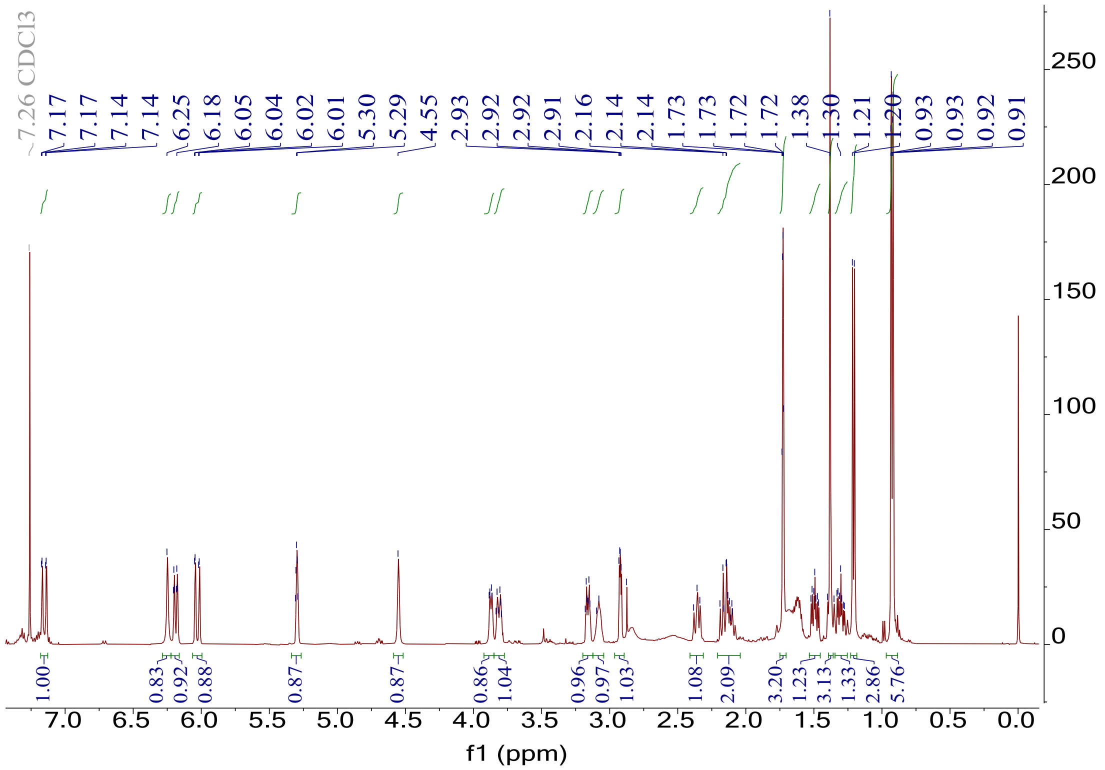

Figure S47. The ^{13}C NMR spectrum of compound **9** in $CDCl_3$.

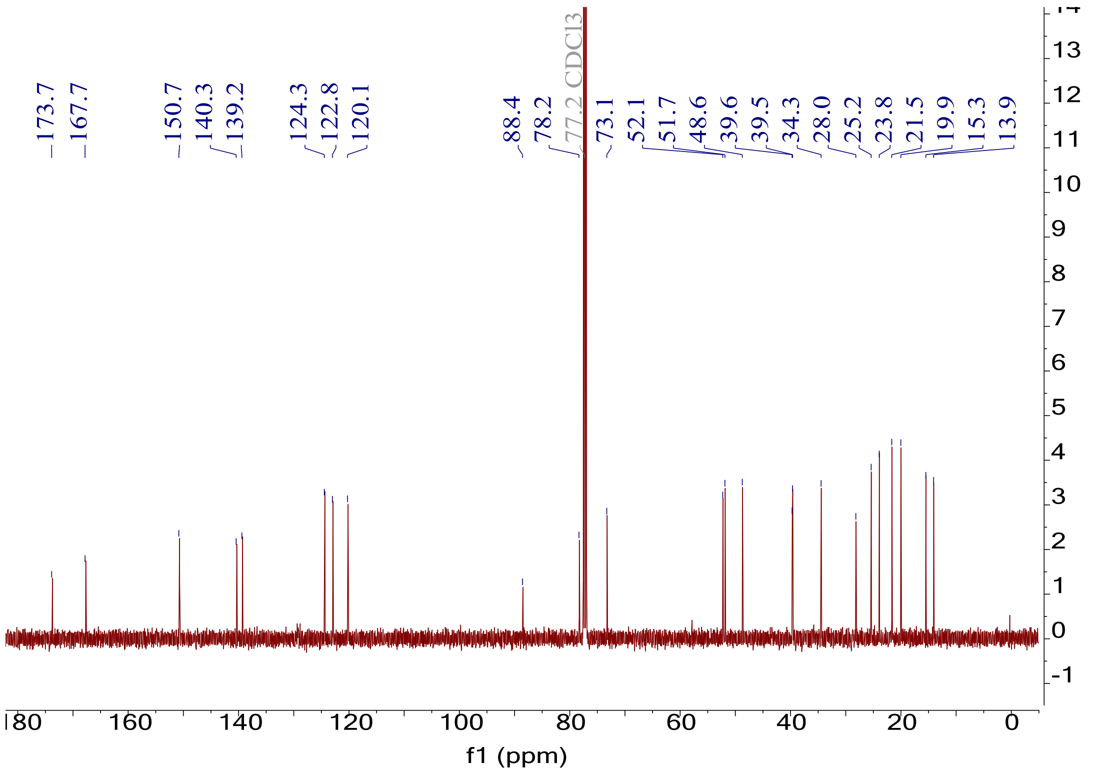

Figure S48. The ^{1}H NMR spectrum of compound **10** in CD_3OD.

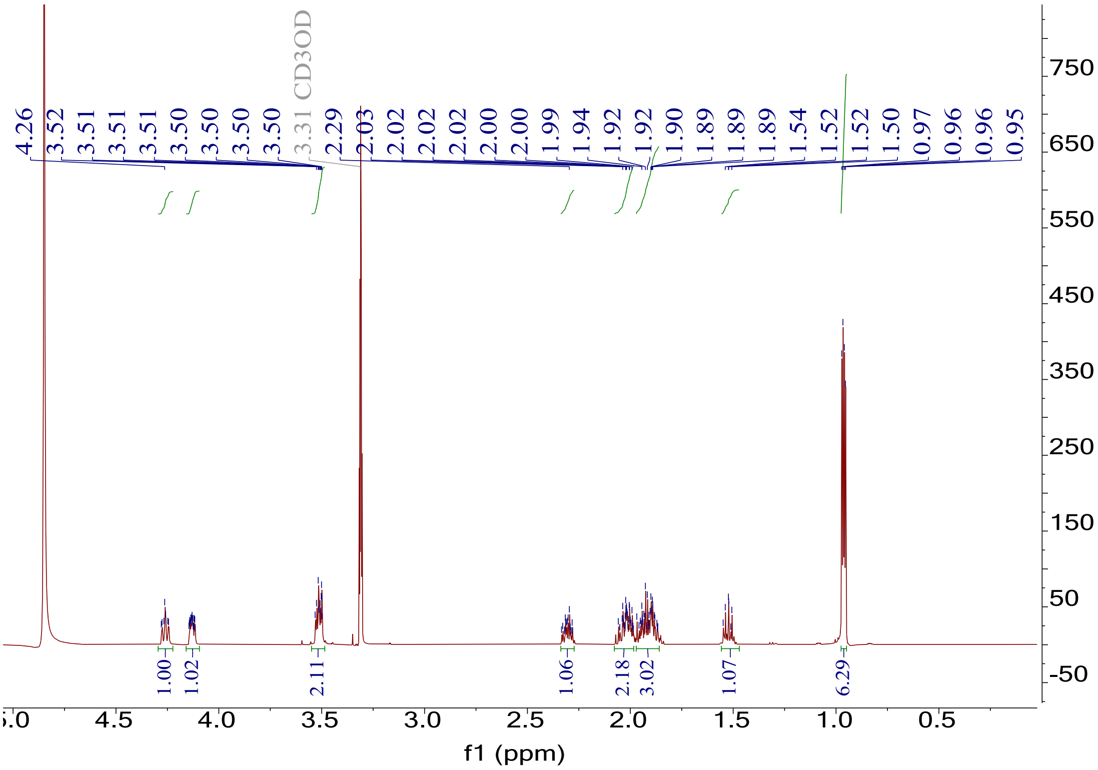

Figure S49. The ^{13}C NMR spectrum of compound **10** in CD_3OD.

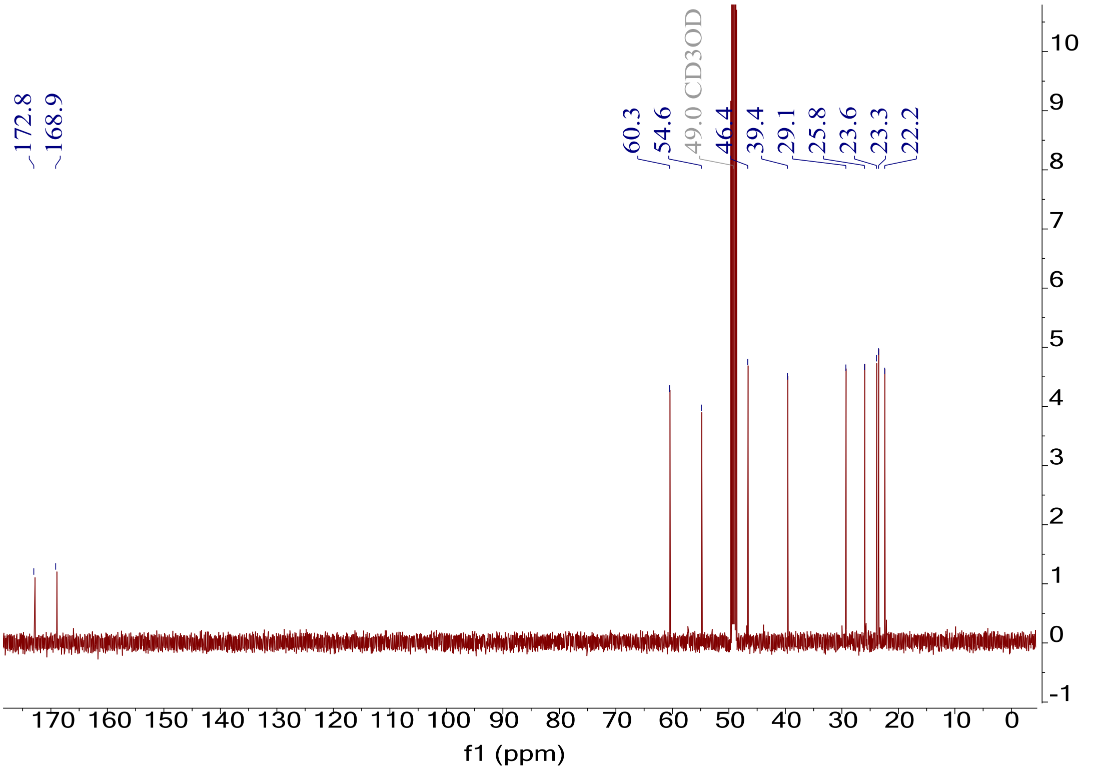

Figure S50. Experimental ECD spectra of **1** and **5**.

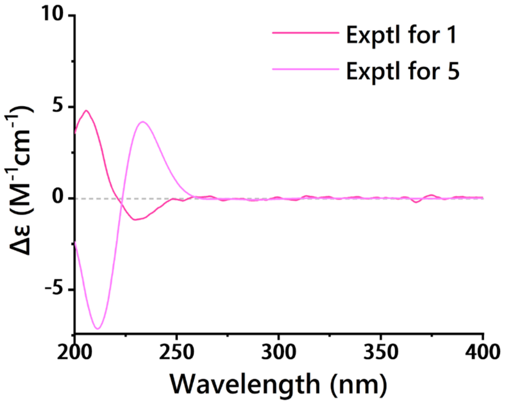

Figure S51. HPLC analysis of Marfey's derivatives of avellanin P (**5**).

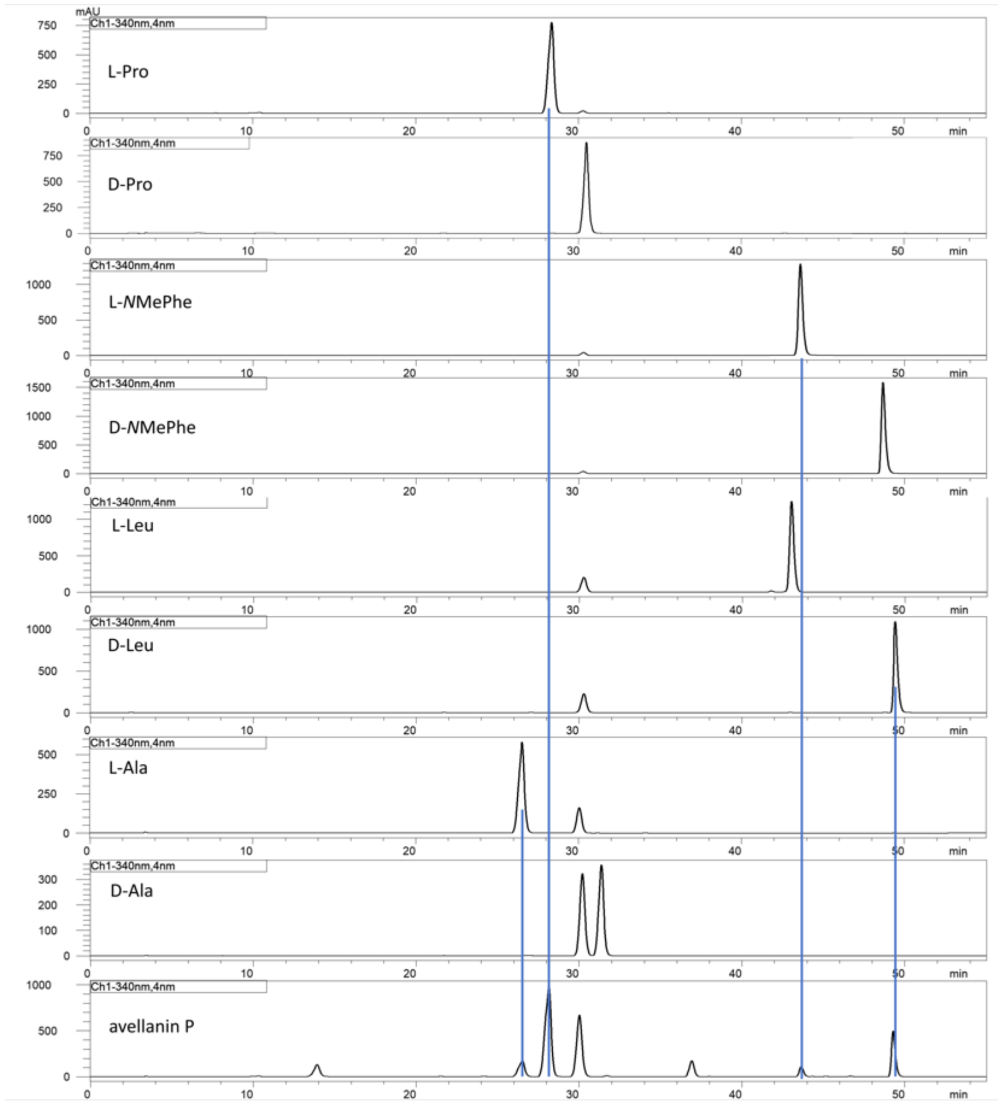

Anti-inflammatory Assays.

Inflammation was induced in the 3-dpf Tg (*zlyz:EGFP*) zebrafish larvae (10 per group) by 1 h exposure to 20 μM $CuSO_4$, which damages the zebrafish neuromasts. Prior to $CuSO_4$ exposure, larvae were pretreated for 2 h with compounds **1**, **2**, **4**, **5** (20 μM), and indomethacin (20 μM). Macrophage migration was quantified by image analysis. Indomethacin was used as the positive control [1].

Cytotoxicity Assays.

The SRB method was used to evaluate cytotoxicity against ASPC-1 (human pancreatic cancer), MDA-MB-231 (human breast cancer), NCI–H446 and NCI–H446/EP (human small cell lung cancer) cell lines. The MTT method was used to evaluate cytotoxicity against the K562 (human leukemia) cell lines. Adriamycin was used as a positive control [2].

References

1. Nguyen, T. H.; Le, H. D.; Nguyen Thi Kim, T.; Pham The, H.; Nguyen, T. M.; Cornet, V.; Lambert, J.; Kestemont, P. Anti–Inflammatory and Antioxidant Properties of the Ethanol Extract of *Clerodendrum Cyrtophyllum* Turcz in Copper Sulfate-Induced Inflammation in Zebrafish. *Antioxidants.* **2020,** 9, 192.
2. Zhang, G.; Yin, R.; Dai, X.; Wu, G.; Qi, X.; Yu, R.; Li, J.; Jiang, T. Design, synthesis, and biological evaluation of novel 7-substituted 10,11-methylenedioxy-camptothecin derivatives against drug-resistant small-cell lung cancer in *vitro* and in *vivo*. *Eur. J. Med. Chem.* **2022,** 241, 114610.
